# Organocatalyzed Asymmetric α-Oxidation, α-Aminoxylation and α-Amination of Carbonyl Compounds

**DOI:** 10.3390/molecules15020917

**Published:** 2010-02-11

**Authors:** Tirayut Vilaivan, Worawan Bhanthumnavin

**Affiliations:** Organic Synthesis Research Unit, Department of Chemistry, Faculty of Science, Chulalongkorn University, Phayathai Road, Patumwan, Bangkok 10330, Thailand; E-Mail: worawan.b@chula.ac.th (W.B.)

**Keywords:** organocatalyst, chiral catalyst, α-oxidation, α-amination, α-aminoxylation, nitrosoaldol reaction, aldehyde, ketone, active methylene compound

## Abstract

Organocatalytic asymmetric α-oxidation and amination reactions of carbonyl compounds are highly useful synthetic methodologies, especially in generating chiral building blocks that previously have not been easily accessible by traditional methods. The concept is relatively new and therefore the list of new catalysts, oxidizing and aminating reagents, as well as new substrates, are expanding at an amazing rate. The scope of this review includes new reactions and catalysts, mechanistic aspects and synthetic applications of α-oxidation, hydroxylation, aminoxylation, amination, hydrazination, hydroxyamination and related α-heteroatom functionalization of aldehydes, ketones and related active methylene compounds published during 2005–2009.

## Outline

1. Introduction
2. α-Amination reactions
  2.1. α-Amination with azodicarboxylate esters
    2.1.1 Simple aldehydes and ketones
    2.1.2 Other carbonyl substrates
    2.1.3. Applications
  2.2. α-Sulfamidation
  2.3. α-Hydroxyamination
3. α-Aminoxylation reactions
  3.1. α-Aminoxylation with nitrosobenzene
    3.1.1. Substrates
    3.1.2. Catalysts
    3.1.3. Applications
    3.1.4. Aminoxylation *vs.* nitrosoaldol reactions
  3.2. α-Aminoxylation with TEMPO
4. α-Oxidation reactions
  4.1. α-Oxybenzoylation
  4.2. α-Oxidation with molecular oxygen
  4.3. α-Oxidation with hydroperoxides
  4.4. α-Aryloxylation with *o*-quinones
  4.5. α-Oxidation with oxaziridine and iodosobenzene
  4.6. α-Oxysulfonation catalyzed by iodoarenes
5. Conclusions and Outlook

## 1. Introduction

**Figure 1:**
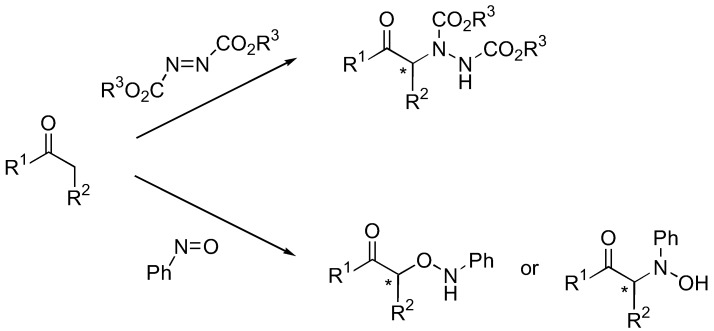
Asymmetric direct α-functionalization of carbonyl with oxygen and nitrogen electrophiles.

The rich chemistry of carbonyl compounds makes optically active α-heteroatom-functionalized carbonyl compounds important precursors for a large group of natural and non-natural compounds possessing a nitrogen or oxygen moiety attached to a stereogenic center. The direct introduction of a nitrogen or an oxygen atom to the α-position of the carbonyl group catalyzed by organocatalysts represents a powerful and promising methodology towards such optically active precursors (Figure 1). 

During the past decade, a number of impressive examples of organocatalytic enantioselective direct α-amination, α-aminoxylation, and α-oxidation reactions on carbonyl compounds to afford the corresponding α-amino and α-hydroxy carbonyl derivatives have been developed. Several reviews on the topic of organocatalyzed direct asymmetric α-aminoxylation [1,2], α-amination [2] as well as α-heterofunctionalization of carbonyl compounds in general [3,4,5,6] or some specific aspects [7,8,9,10,11] exist up to the year 2006. This review compiles a significant progress made up to the year 2009.

## 2. α-Amination Reactions

In 2002, Jørgensen [12] and List [13] independently reported the first successful proline-catalyzed enantioselective direct α-amination of aldehydes using azodicarboxylate esters as electrophiles. Since then, a number of α-aminations of simple and branched aliphatic aldehydes, as well as aliphatic cyclic and acyclic ketones catalyzed by proline and its derivatives have been reported. Early works have been covered in previous reviews on this subject [2,3,4,5]. Analogous reactions with other substrates such as 1,3-dicarbonyl compounds or α-substituted-α-cyanoacetates have been accomplished with different catalysts, including those derived from cinchona alkaloids [8], binaphthyl [14,15,16], or cyclohexanediamine derivatives [17]. Details of recent developments in the above mentioned topics will be described herein.

### 2.1. α-Amination with Azodicarboxylate Esters

#### 2.1.1. Simple aldehydes and ketones

The α-amination of simple α-unbranched aldehydes with various azodicarboxylate esters proceeds very efficiently under catalysis by L-proline (**1**, Table 1). Since the α-aminated products are often chemically and configurationally unstable due to the presence of an acidic α-H, they are often reduced *in situ* to the corresponding aminoalcohols or, after subsequent cyclization under basic conditions, to *N*-aminooxazolidinones [12,13,14,18,19,20,14,18].

**Table 1:** Proline catalyzed α-amination of unbranched aldehydes. 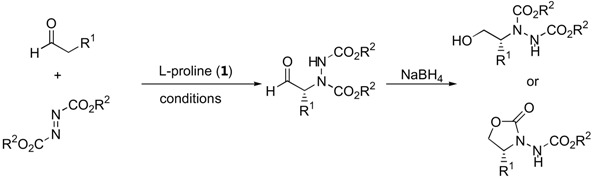

R^1^	R^2^	Conditions	Yield (%)	*ee* (%)	Ref.
Me, Et, ^i^Pr, *^t^*Bu, allyl, Bn	Et, Bn	10 mol% **1**, CH_2_Cl_2_, RT	57–92	89–95	[12]
Me, ^i^Pr, *^n^*Pr, *^n^*Bu, Bn	Bn	10 mol% **1**, CH_3_CN, 0 °C to RT, 3h	93–99	>95	[13]

In later reports, the possibility of generating quaternary stereogenic centers at the α-position of α-branched aldehydes under catalysis by proline (**1**) [21,22], pyrrolidinyl tetrazole (**2**) [23,24] or L-azetidine-2-carboxylic acid (**3**) [21] were explored (Table 2). The observed enantioselectivities ranged from essentially none to >99%. Derivatives of 2-phenylpropanal gave better enantioselectivities than α,α-dialkyl substituted aldehydes.

**Table 2:** α-Amination of α-branched aldehydes catalyzed by proline and derivatives. 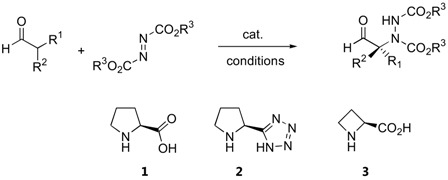

R^1^	R^2^	R^3^	Conditions	Yield (%)	*ee* (%)	Ref.
Et, *^n^* Bu, Ph, 2-Naph,	Me,	Et,	50 mol% **1**,	17­–87	4–28 (R^1^ = alkyl)	[21]
subst. Ph	Et	Bn	CH_2_Cl_2_, RT,		76–85 (R^1^ = aryl)	
			2.5–9 d			
Et, Ph, 2-Naph,	Me,	Et,	50 mol% **3**,	N/A	6 (R^1^ = alkyl)	[21]
subst. Ph	Et	Bn	CH_2_Cl_2_, RT,		49–78 (R^1^ = aryl)	
			2.5–9 d			
Me, Et, Pr, Bu, Ph,	Me,	Et,	50 mol% **1**,	19–99	0–39 (R^1^ = alkyl)	[22]
2-Naph, subst. Ph,	Et	Bn	CH_2_Cl_2_, RT		35–86 (R^1^ = aryl)	
2-thienyl						
H, 2-Naph,	Me	Et	50 mol% **1** or **2**,	54–97	52–91	[24]
4-subst. Ph			MeCN, microwave	(6–85)^a^	(24–84)^a^	
4-BrC_6_H_4_CH_2_	Me	Bn,	15 mol% **2**, MeCN,	95	80	[23]
		Et	RT, 3h	(90)^b^	(44)^b^	
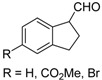		Bn	20–30 mol% **1** or *ent*-**1**, MeCN, RT,1–4 h	75–99	>99	[25]

^a^ Without microwave irradiation ^b^ With 30 mol% L-proline (**1**) instead of **2** as catalyst.

Bräse and coworkers had employed microwave irradiation to accelerate the rate of proline-catalyzed amination, and found that yields as well as enantioselectivity had somewhat improved after shorter reaction times [24]. It appears that the pyrrolidinyl tetrazole **2** was a more effective catalyst for amination of 2-phenylpropanal derivatives than L-proline (**1**) [23,24]. Indane-1-carboxaldehydes are particularly good α-branched aldehyde substrates for L-proline- (**1**) or D-proline (*ent*-**1**)-catalyzed α-amination [25]. The corresponding amination products were obtained in 99% and >99% *ee* and were subsequently elaborated into the metabotropic glutamate receptor ligands, (*S*)-AIDA and (*S*)-APICA[25]. The possibilities of obtaining good yields and enantioselectivities from these inherently chiral aldehydes suggests that the original chirality of the aldehyde has no effect on the stereoselectivity, which is to be expected based on the proposed mechanism that proceeds through planar intermediates.

**Figure 2:**
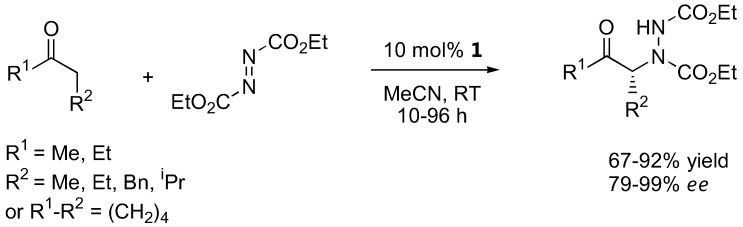
L-Proline-catalyzed α-amination of ketones [26].

Jørgensen and coworkers also reported a proline-catalyzed enantioselective direct α-amination of ketones (Figure 2) [26]. The reaction of simple aliphatic ketones with diethyl azodicarboxylate (DEAD) was found to be fairly regioselective as the amination took place at the more highly substituted carbon atom (ratio = 76:24–91:9). The reaction also proceeded with excellent enantioselectivities. Subsequent reduction and cyclization could be carried out to afford the *syn*-α-amino alcohols or the *N*-amino-oxazolidinones [26]. 

**Table 3:** α-Amination of cyclohexanone and derivatives. 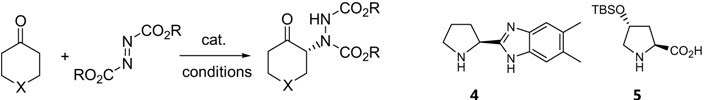

ketone	R	cat.	conditions	yield^a^ (%)	*ee*^b^ (%)	ref.
X=CH_2_	Bn	**3**	20 mol% cat., CH_2_Cl_2_, 25 ºC , 24 h	60 (56)	90 (61)	[27]
X=CH_2_	Bn	**4**	10 mol% cat./TFA, CH_2_Cl_2_, 20 ºC, 24 h	92 (88)	66 (84)	[28]
X=SiPh_2_	Bn	**4**	20 mol% cat./TFA, CH_2_Cl_2_, 20 ºC, 24 h	65 (85)	71 (60)	[28]
X=CH_2_	Et	**5**	10 mol%, ClCH_2_CH_2_Cl, RT, 1.5–3 h	89 (31)	85 (85)	[29]
X=CH_2_	Bn	**5**	10 mol%, ClCH_2_CH_2_Cl, RT, 1.5–3 h	86 (50)	94 (75)	[29]

^a,b^ Numbers in parenthesis are results from L-proline catalyzed reactions under comparable conditions.

Cyclic ketones such as cyclohexanone and derivatives generally gave poor results with proline catalyst **1** (Table 3). The α-amination of cyclohexanone with DEAD or dibenzyl azodicarboxylate (DBAD) under L-azetidine-2-carboxylic acid (**3**) catalysis showed improved enantioselectivities of 88–90% [27]. The benzimidazole-pyrrolidine (BIP) catalyst **4 **was comparable to L-proline in terms of enantioselectivity with cyclohexanone and 1-silacyclohexan-4-one as substrates [28]. In a comparison between the efficiency of L-proline (**1**) versus 4-silyloxyproline (**5**) in the α-amination of cyclohexanone and derivatives, the catalyst **5** provided higher yields and enantioselectivities than **1** [29]. 

**Figure 3:**
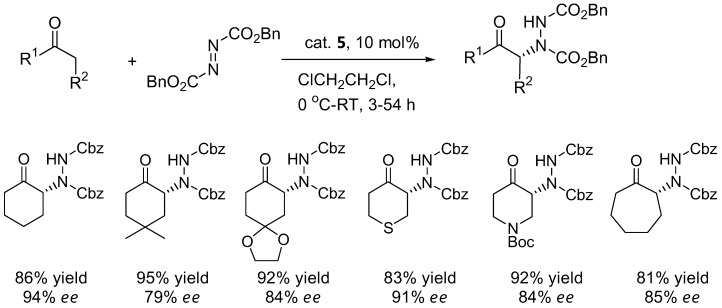
Examples of products from α-amination of cyclic ketones catalyzed by the silyloxyproline **5** [29].

The silyloxyproline catalyst **5** proved to be a quite general catalyst for amination of ketones. In addition to cyclohexanone derivatives (Figure 3), the catalyst can also accept acyclic ketones and α-branched aldehydes as substrates (64–73% yield, 78–96% *ee*) [29]. 

**Figure 4:**
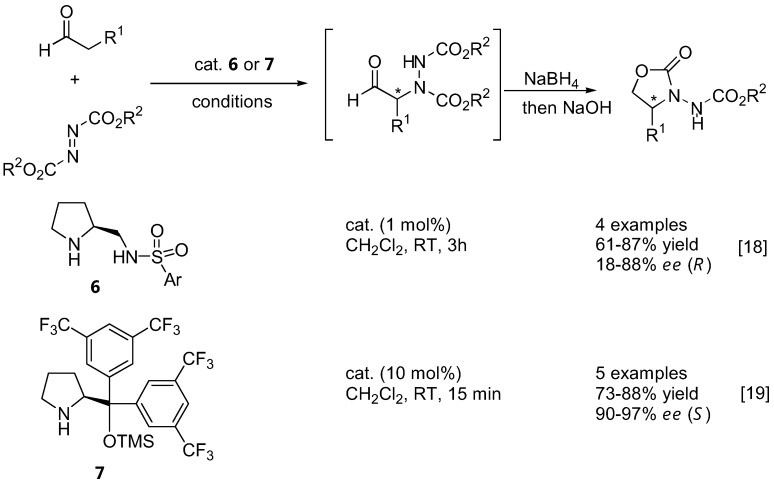
Additional non-amino acid catalysts for α-amination of aldehydes.

Ionic liquids have been used as alternative solvent for L-proline-catalyzed α-amination of aldehydes and ketones [30]. Unfortunately, both the product yields and enantioselectivities were not comparable to those obtained from reactions in conventional solvents. The poor results may partly be due to side reactions between the ionic liquid [bmim]BF_4_ and the aminating agent (DEAD) used.

**Figure 5:**
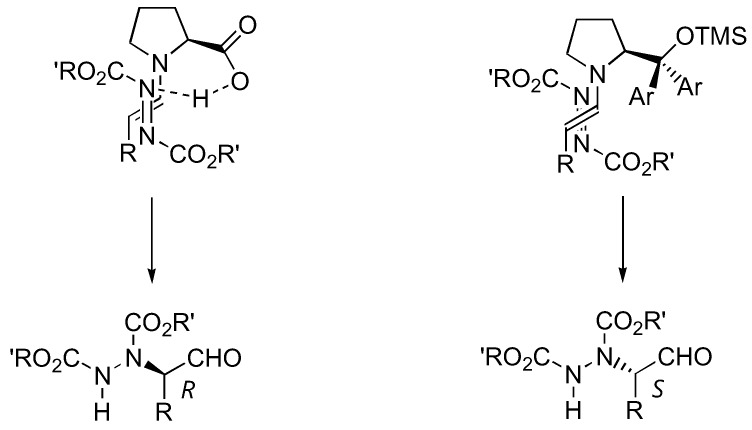
Models to explain the opposite enantioselectivity of amination of aldehydes catalyzed by **1** and **7**.

The aforementioned catalysts **1**–**5**, as well as the sulfonamide catalyst **6 **[18] (Figure 4), are based upon the proline skeleton with a hydrogen bond donating substituent. Such catalysts operate through the dual activation of both the aldehyde (by forming a more nucleophilic enamine) and the electrophile (by hydrogen bonding). It is generally believed that the approach of the electrophile (azodicarboxylate ester in this case) was controlled by hydrogen bonding with the carboxyl group of the catalyst. On the other hand, the presence of a hydrogen-bonding group was not prerequisite to high activities and enantioselectivities. Jørgensen *et al.* have developed a new silyl-protected diarylprolinol catalyst **7** that could not participate in hydrogen bonding [19] (Figure 4). The enantioselectivities observed with this catalyst are thus originated from controlling the geometry of the enamine and from steric bias of the two enamine faces by the large substituent on the pyrrolidine ring of the catalyst. This catalyst proved to be highly active, and α-amination of aldehydes with DEAD or di-isopropyl azodicarboxylate (DIAD) in the presence of 10 mol% of **7** was completed in 15 min at room temperature, giving the product in 73–88% yield and 90–97% *ee* [19]. Most importantly, despite the same absolute configuration of the catalyst **7** as that of proline, the reactions catalyzed by them afforded opposite enantiomers of the same product. This could be explained by two different transition state models shown in Figure 5. The origin of the stereoselectivity was further investigated by DFT calculations [31].

**Figure 6:**
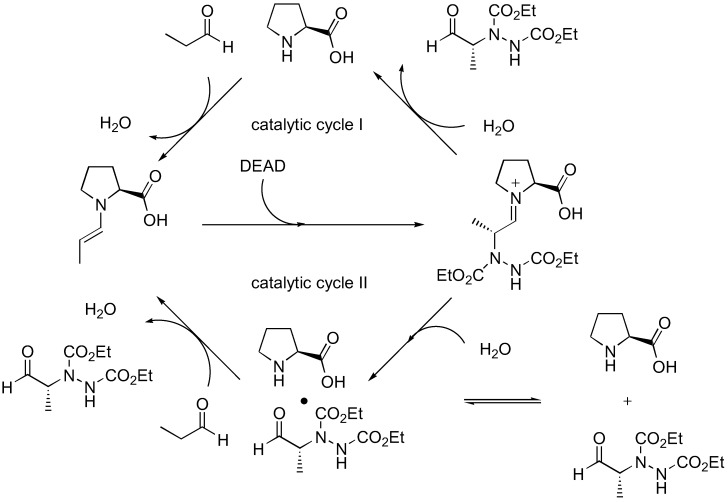
Catalytic cycles of proline-catalyzed amination of aldehydes as proposed by Blackmond *et al.* [34].

Although this simple model can satisfactorily explain the observed enantioselectivity of proline-catalyzed α-amination of aldehydes, it cannot account for the autoinductive behavior and asymmetric amplification observed upon detailed kinetic analysis by Blackmond *et al.* [32,33]. Such unusual kinetics for proline-catalyzed reactions are observed only in amination and aminoxylation (Section 3.1.2), but not aldol reactions. The possibility of rate acceleration by solubilization of the proline catalyst over time was ruled out since a proline derivative that is completely soluble also displays the same kinetic profile [34]. An alternative catalytic cycle involving product catalysis that is consistent with the kinetic data was proposed (Figure 6) [34].

#### 2.1.2. Other carbonyl substrates

##### Aromatic ketones

Proline and its derivatives are generally not effective catalysts for α-amination as well as other electrophilic reactions of aromatic ketones. Recently, a successful enantioselective direct α-amination of aromatic ketones catalyzed by primary amines derived from cinchona alkaloids has been reported [35]. 

**Figure 7:**
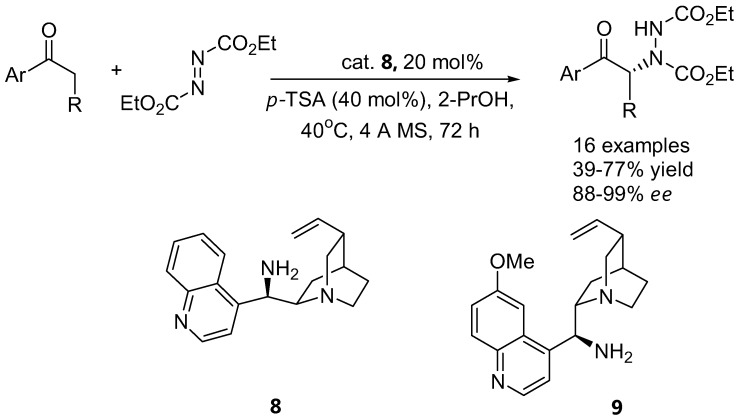
Direct α-amination of aromatic ketones catalyzed by primary amines derived from cinchona alkaloids [35].

In most cases, excellent enantioselectivities were observed for various aromatic ketones in the presence of the catalyst 9-amino-9-deoxyepicinchonine (ADC, **8**) (Figure 7). The use of 9-amino-9-deoxyepiquinine (ADQ, **9**) as catalyst afforded the opposite enantiomer of the α-aminated product in lower enantiomeric purity and lower yield. It was proposed that activation of the carbonyl with a primary amine would experience less steric interaction than with a secondary amine. In general the reaction was also found to be quite sensitive to steric groups on the azodicarboxylate electrophiles, for instance DEAD was found to give better enantiomeric excess than DBAD. A catalytic model of the reaction was also proposed to account for the observed stereochemistry of the product [35].

##### Dicarbonyl compounds

**Figure 8:**
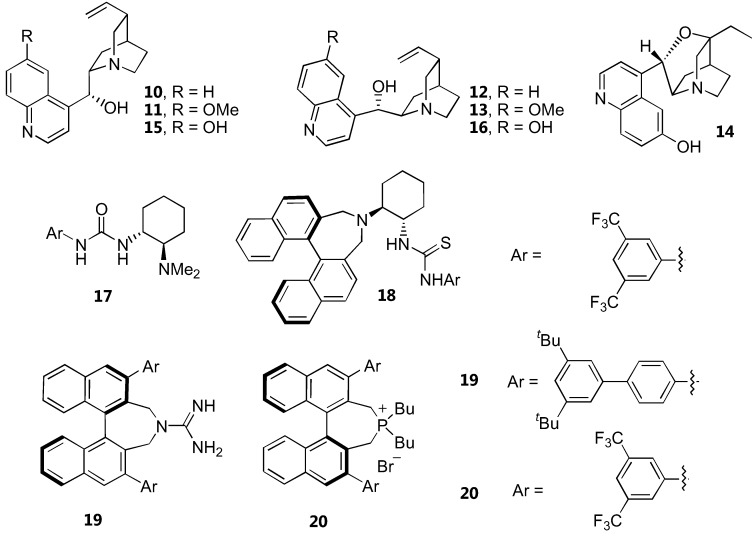
Various catalysts for direct α-amination of 1,3-dicarbonyl compounds.

Structures of catalysts for α-amination of 1,3-dicarbonyl compounds are shown in Figure 8. The yields and enantioselectivities are summarized in Table 4. Simple unmodified cinchona alkaloids, cinchonidine (**10**) and cinchonine (**12**), have reportedly catalyzed α-amination of cyclic β-ketoesters in high yields and good enantioselectivity [36]. High reaction rates and good enantioselectivities were observed with five membered β-ketolactones and ketoesters. This was rationalized by the higher reactivity of the resulting enolate. In contrast, acyclic β-ketoesters required longer reaction time and gave only moderate to poor enantioselectivity (<40% *ee*). The structurally similar quinine (**11**) and quinidine (**13**) gave much lower enantioselectivities which could be attributed to the basicity or conformation difference.

**Table 4:** Enantioselective α-amination of 1,3-dicarbonyl compounds with various catalysts. 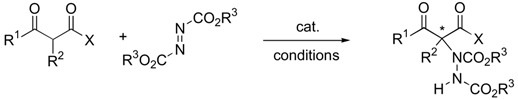

Cat.	1,3-Dicarbonylcompounds	Conditions	Yield(%)	*ee*(%)	Ref.
**10**	acyclic β-keto esters	20 mol% cat., -25 ºC, 7 d	72	47	[36]
**12**	acyclic β-keto esters	20 mol% cat., -25 ºC, 7 d	72	27	[36]
**10**	cyclic β-keto esters	20 mol% cat., -25 ºC, 2 min – 48 h	92–95	54–88	[36]
**12**	cyclic β-keto esters	20 mol% cat., -25 ºC, 5 min – 24 h	81–95	77–87	[36]
**10**	cyclic β-keto lactones	20 mol% cat., -25 ºC, 2 min – 4 d	68–91	49–60	[36]
**12**	cyclic β-keto lactones	20 mol% cat., -25 ºC, 2 min – 4 d	51–96	42–57	[36]
**14**	acyclic and cyclic β-keto esters	5 mol% cat., -52ºC –RT, toluene, 16–143 h	86–99	83–90	[37]
**15 or 16**	acyclic β-keto thioesters	0.5–10 mol% cat., -78 ºC to RT, 5 min–3 h	91–95	90–>99	[38]
**17**	cyclic β-keto esters	10 mol% cat., toluene, -78 ºC to RT, 0.5–48 h	84–98	50–92	[17]
**18**	cyclic β-keto esters	10 mol% cat., toluene, -70 to -30ºC	85–95	93–99	[14]
**19**	cyclic β-keto esters	0.05–2 mol% cat., THF, -60 ºC, 0.5–24 h	>99	97–98	[15]
**19**	acyclic β-keto esters	2 mol% cat., THF, -60 ºC, 0.5–24 h	54–>99	62–85	[15]
**19**	cyclic β-diketones	2 mol% cat., THF, -60 ºC, 0.5–24 h	≥99	15–91	[15]
**20**	cyclic β-keto esters	3 mol% cat., K_2_CO_3_ or K_2_HPO_4_, toluene, -20 to -40 ºC, 2–96 h	97–99	77–95	[16]
**20**	*tert*-butyl α-fluoro-benzoylacetate	3 mol% cat., K_2_HPO_4_, toluene, -20 ºC, 84 h	99	73	[16]
**20**	functionalized cyclic diketone	5 mol% cat., K_2_HPO_4_, toluene, -20 ºC, 84 h	75	88	[16]

Another compound in the cinchona alkaloid family, β-isocupreidine (β-ICD, **14**), could also enantioselectively catalyze α-amination of 1,3-dicarbonyl compounds [37]. The reaction of α-substituted 1,3-dicarbonyl compounds including cyclic and acyclic β-ketoesters and 1,3-diketones with di-*tert*-butyl azodicarboxylate proceeded with high conversion and enantioselectivity.

Deng and coworkers [38] proposed that the low yields and poor enantioselectivities observed in the asymmetric α-amination of the less reactive acyclic β-ketoesters catalyzed by cinchona alkaloids may result from catalyst inactivation *via* a Friedel-Crafts type reaction with the diazocarboxylate esters. It was therefore envisaged that β-ketothioesters bearing a more acidic α-proton than simple β-ketoesters might be sufficiently reactive to undergo the amination before the catalyst inactivation. Gratifyingly, the α-amination reaction of various acyclic and cyclic β-ketothioesters proceeded very well in the presence of the catalysts **15** or **16 **to afford the products with high yields and enantiomeric excesses.

A chiral bifunctional urea 17 derived from cyclohexanediamine had been proposed as alternative catalyst for α-amination of cyclic β-ketoesters [17]. The reactions of cyclic β-ketoesters gave excellent yields and high enantioselectivity. A related chiral diamine-thiourea derivative derived from a binaphthyl scaffold 18 has been developed as a catalyst for α-amination of β-ketoesters [14]. Although the α-amination of cyclic β-ketoesters proceeded efficiently giving high yields and very high enantioselectivity, the acyclic counterparts underwent the reaction very slowly, giving very low enantiomeric excess in the products. A transition state involving the electrophile activation by hydrogen bonding with the thiourea and the β-ketoester activation by the basic nitrogen atom in tertiary amine has been proposed for the catalyst 18. A DFT calculation confirmed that the catalyst 17 deprotonates the β-ketoester and binds the two substrates through hydrogen bonding with the urea and the protonated amino groups [39].

The axially chiral guanidine **19**, which should possess a *C*_2_ symmetry when protonated, is another highly active catalyst for α-amination of various cyclic and acyclic 1,3-dicarbonyl compounds [15]. The chiral phosphonium salt **20** derived from the same binaphthyl skeleton was also a very effective catalyst for α-amination of a range of 1,3-dicarbonyl compounds. One particularly interesting aspect of this catalyst is that it can accept functionalized substrates such as α-fluorobenzoylacetate ester [16].

##### α-Substituted α-cyanoacetates and α-cyanoketones

In 2004, Jørgensen *et al.* reported the first organocatalytic α-amination of α-substituted α-cyanoacetates [37]. The cinchona alkaloid β-isocupreidine (**14**), which has been successfully used for α-amination of 1,3-diketones, was also an equally effective catalyst for α-amination of a range of α-aryl α-cyanoacetates [37]. The cinchona alkaloid-derived catalysts **15** and **16** were effective for α-aryl α-cyanoacetates [40] and α-alkyl α-cyanoacetates [38]. In the latter case, a more acidic 2,2,2-trifluoroethyl ester was required to achieve good enantioselectivity [38]. Other natural alkaloids and synthetic chiral amines such as **21­**–**23** have also been reported as catalysts for α-amination of ethyl α-phenyl α-cyanoacetate [41,42]. However, the enantioselectivities were not as high as cinchona alkaloid derivatives and substrate generality has not been demonstrated. The results on direct α-amination of α-substituted α-cyanoacetates are summarized in Table 5.

Amination of α-cyanoketones by di-*tert*-butyl azodicarboxylate has also been investigated. The thiourea catalyst **18** (1–10 mol%, toluene -78 °C to RT, 0.5 h–8 d) was found to give the best results. Various cyclic (five- and six-membered ring) and acyclic cyanoketones were excellent substrates for this reaction (14 examples, 45–95% yield, 87–99% *ee*) [43]. On the other hand, this catalyst gave only moderate enantioselectivity in direct amination of ethyl α-phenyl α-cyanoacetate (up to 65% *ee*) [43].

**Table 5:** Direct α-amination of α-substituted α-cyanoacetates. 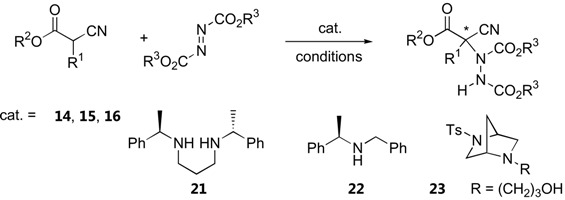

Cat.	R^1^	R^2^	R^3^	Conditions	Yield (%)	*ee*(%)	Ref.
**14**	Ph, subst. Ph, 2-Naph, 2-thienyl	*^t^*Bu	*^t^*Bu	5 mol% cat., toluene, -50 to -78 °C, 16–20 h	95–99	89–>98	[37]
**15**	Ph, subst. Ph,1-Naph	Et	*^t^*Bu, Bn	5–10 mol% cat., -78 °C, toluene, 1 min–12 h	72–98	87–99	[40]
**16**	Ph, subst. Ph, 1-Naph	Et	*^t^*Bu, Bn	5–10 mol% cat., -78 °C, toluene, 1 min–12 h	71–99	82–95	[40]
**15**	Me	CF_3_CH_2_	*^t^*Bu	10 mol% cat., toluene, -60 °C	99(74)^a^	91(23)^a^	[38,40]
**16**	Me	CF_3_CH_2_	*^t^*Bu	10 mol% cat., toluene, -60 °C	99(75)^a^	81(35)^a^	[38,40]
**18**	Ph	Et	Et	10 mol% cat., toluene, RT, 1 h	86	65	[43]
**21**	Ph	Et	*^t^*Bu	50 mol% cat., 100 °C, 1:1 toluene:hexane, 30 min	91	84( *R*)	[41]
**22**	Ph	Et	*^t^*Bu	1–50 mol% cat., -100 °C 1:1 toluene:hexane, 0.5-20 h	90–95	64–80( *S*)	[41]
**23**	Ph	Et	*^t^*Bu	25 mol% cat., toluene, -78 °C, 24 h	76	40( *R*)	[42]

^a^ Yield and enantioselectivities obtained with ethyl α-methyl α-cyanoacetate (R^2^ = Et).

##### Miscellaneous substrates

Direct aminations of chiral protected β-hydroxyaldehydes under catalysis by proline have been recently reported by Greck [44]. The configuration of the newly formed stereogenic center at the α-carbon was mainly controlled by the catalyst, although some matched-mismatched effects have been observed. The 3- and 4-*tert*-butoxy derivatives of proline catalysts developed by the same group [45] gave comparable results to unmodified proline.

**Figure 9:**
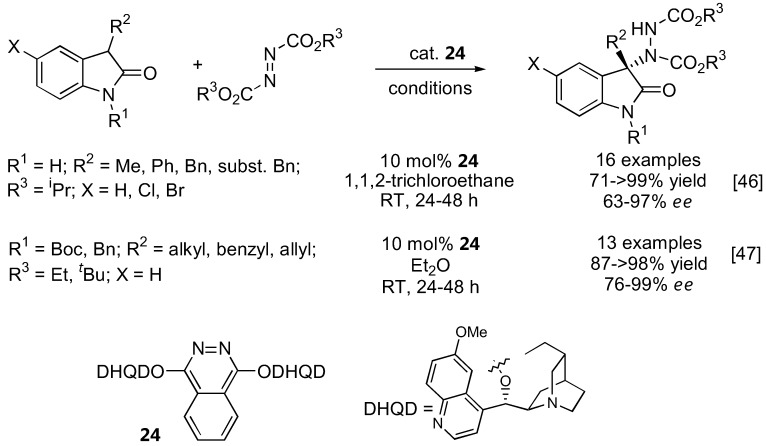
Direct α-amination of 3-substituted 2-oxindoles catalyzed by **24 **[46,47].

Two independent reports appeared in 2009 describing the first α-amination of 3-substituted 2-oxindoles under catalysis by a cinchona alkaloid-derived catalyst (Figure 9) [46,47]. Although the same (DHQD)_2_PHAL catalyst **24** was used, the substrate scopes are somewhat different, presumably due to the different conditions employed. The free indole NH was initially thought to be essential to the reactivity as well as enantioselectivity [46]. However, it was later demonstrated that a variety of *N*-substituted oxindoles also gave excellent yield and enantioselectivities [47].

**Figure 10:**
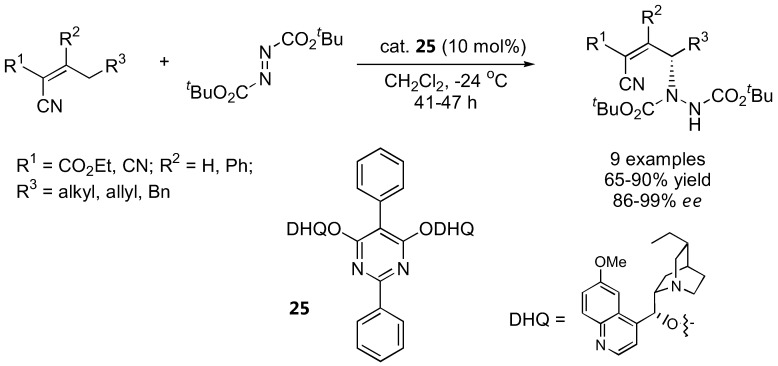
Direct allylic amination of alkylidene cyanoacetates catalyzed by **25 **[49].

Although not strictly an α-amination of carbonyl compounds, it is worth including the two recently reported amination at the γ-positions of α,β-unsaturated aldehydes [48] and alkylidene cyanoacetates [49], both from the Jørgensen group. Cinchona alkaloids were employed in the allylic amination of alkylidene cyanoacetates (Figure 10) [49]. 

**Figure 11:**
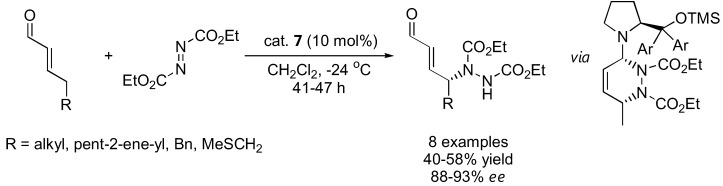
Direct γ-amination of α,β-unsaturated aldehydes catalyzed by **7** [48].

The (DHQ)_2_PYR catalyst **25** gave the best results. The highly functionalized γ-hydrazino alkylidene cyanoacetates could be further transformed into a variety of products [49]. For γ-amination of α,β-unsaturated aldehydes, the silyl-protected diarylprolinol (**7**) was more effective than proline and other derivatives (Figure 11) [48]. The stereochemistry of the γ-amination product was opposite to that of the α-amination of simple aldehydes using the same catalyst. A hetero-Diels-Alder reaction between the (*E*,s-*cis*,*E*)-dienamine intermediate and the azodicarboxylate ester was proposed based on DFT calculations to account for the unexpected stereochemical outcome [48]. 

#### 2.1.3. Applications

**Figure 12:**
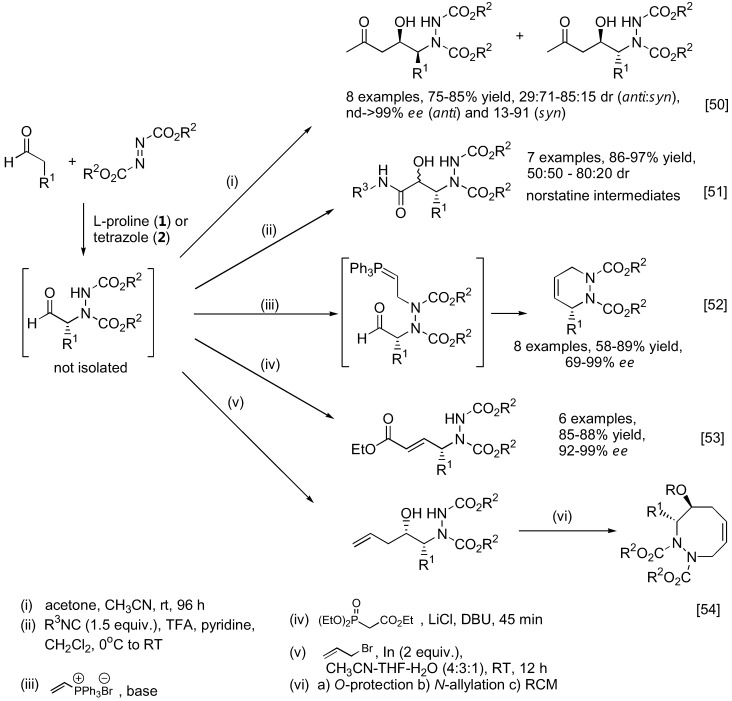
Transformation of α-hydrazinoaldehydes to more complex products or intermediates.

**Figure 13:**
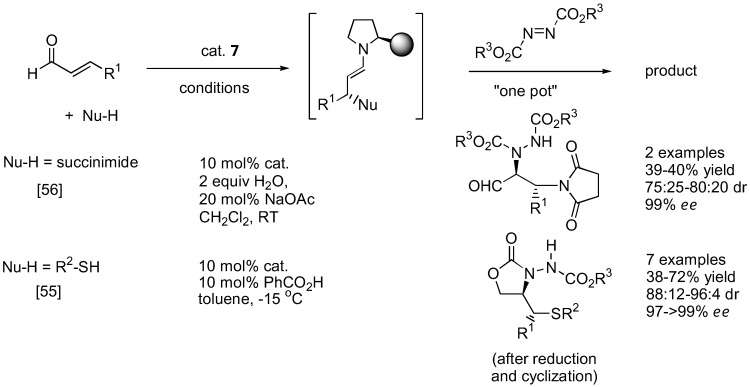
Tandem hetero-Michael addition-α-amination of α,β-unsaturated aldehydes [55,56].

As mentioned earlier, the unstable α-hydrazinoaldehydes are often not isolated but rather subjected to further reactions such as reduction to yield a more stable amino alcohol or reduction-cyclization to afford the corresponding oxazolidinone. Other transformations are illustrated in Figure 12. For example, the so-formed α-hydrazinoaldehyde can further react with acetone under catalysis by **1** in a one-pot fashion to give optically active β-amino alcohols containing two stereogenic centers [50]. A subsequent Passerini reaction of the α-hydrazinoaldehyde may be performed by reacting the aldehyde with an isocyanide. Schmidt *et al.* had shown that this sequential reaction can provide rapid access to norstatine-based peptidomimetics [51]. A tandem α-amination followed by *N*-alkylation with a vinyl phosphonium salt gave a transient ylid intermediate which would then cyclize through an intramolecular Wittig reaction to furnish chiral 3,6-dihydropypridazines [52]. Alternatively, the α-hydrazino aldehyde can be trapped with the Horner-Wadsworth-Emmons (HWE) reagent (triethyl phosphonoacetate) under basic conditions to afford γ-hydrazino-α,β-unsaturated esters [53]. Applications of this one-pot α-amination-olefination of aldehydes in the synthesis of chiral 2-pyrrolidones have been demonstrated. Functionalization of the so-formed α-hydrazino aldehyde by a one-pot indium-promoted allylation followed by a ring-closing metathesis (RCM) would lead to an enantioselective synthesis of cyclic 8-membered ring hydrazines [54]. To further expand the scope of the α-amination reaction of aldehydes, it was demonstrated that β-functionalized aldehydes may be obtained from hetero-Michael type addition to α,β-unsaturated aldehydes under iminium catalysis by the silylprolinol catalyst **7** (Figure 13) [55,56]. The so-formed enamine intermediate may then undergo a subsequent α-amination to afford densely functionalized aldehydes bearing two stereogenic centers. Both sulfur [55] and nitrogen [56] heteroatom nucleophiles have been successfully used in combination with azodicarboxylate esters as the aminating agent in good yield, diastereo- and enantio-selectivities.

**Figure 14:**
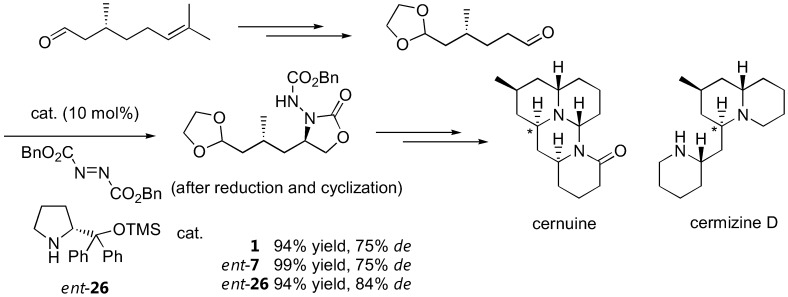
Total synthesis of cernuine and cermizine D incorporating proline-catalyzed α-amination as a key step. The stereogenic centers created by the α-amination are marked by an asterisk [20].

There are numerous applications of proline-catalyzed α-aminations in the synthesis of bioactive compounds. Parts of these have been previously reviewed [57]. For example, L-proline (**1**)-catalyzed α-amination has been shown as a key step in the synthesis of (*R­*)-pipecolic acid and D-proline in moderate to good yields and good enantioselectivity [58]. The D-proline-derived catalyst *ent*-**7 **and its analogue *ent*-**26** have also been reported to induce diasteroselectivity in the 75–84% range when applied to α-amination of a (+)-citronellal-derived aldehyde as part of the lycopodium alkaloids cernuine and cermizine D synthesis [20] (Figure 14). Another application of organocalalyzed enantioselective α-amination in the synthesis of biologically active molecules was elaborated by Barbas III [23]. As a key step in the synthesis of BIRT-377, a potent inhibitor of the interaction between intercellular adhesion molecule-1 (ICAM-1) and lymphocyte function-associated antigen-1 (LFA-1), the pyrrolidinyl tetrazole (**2**) was employed as the α-amination catalyst [23]. Two more recent applications include the syntheses of intermediates for (-)-anisomycin [59] and fumimycin [60], an antibacterial peptide deformylase inhibitor.

### 2.2. α-Sulfamidation

**Figure 15:**
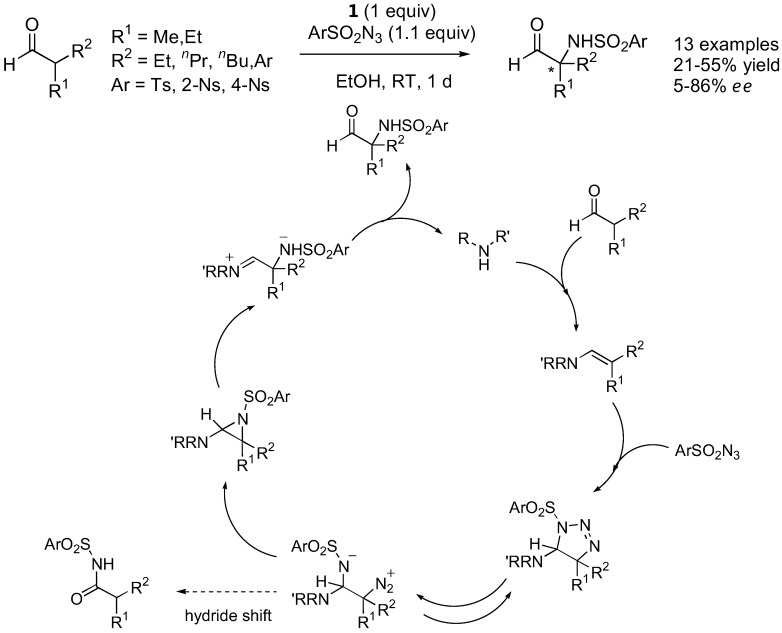
α-Sulfamidation of α-branched aldehydes catalyzed by L-proline (**1**) [61].

Tosyl and nosyl azides reacted with α-branched aldehydes under catalysis by L-proline (**1**) to give the configurationally stable α-sulfamidated aldehydes in *ee* ranging from poor to fairly good (up to 86%) [61]. Interestingly, α-unbranched aldehydes provided *N*-tosylamides instead of the desired sulfamidated products. The mechanism was proposed to involve a 1,3-dipolar cycloaddition pathway (Figure 15).

### 2.3. α-Hydroxyamination

Organocatalytic hydroxyamination of aldehydes and ketones may take place using nitrosobenzene as the aminating agent. The selectivity between hydroxyamination (nitrosoaldol) and aminoxylation reactions is determined both by the substrate and the catalyst. For detailed discussion see Section 3.1.4 - α-Aminoxylation *vs.* nitrosoaldol reactions.

## 3. α-Aminoxylation Reactions

### 3.1. α-Aminoxylation with nitrosobenzene

#### 3.1.1. Substrates

**Figure 16:**
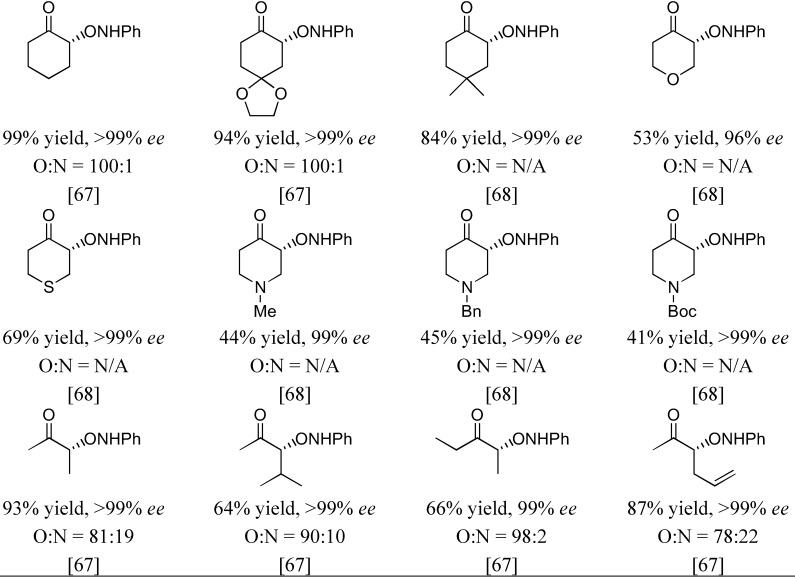
Examples of aminoxylated ketones obtained from L-proline (**1**) catalysis [67,68].

The first organocatalyzed enantioselective aminoxylation of aldehydes were independently reported by Hayashi [62], MacMillan [63], and Zhong [64] in 2003. Simple α-unbranched aliphatic aldehydes reacted with nitrosobenzene under proline catalysis by the attack at oxygen to give exclusively the α-aminoxylated aldehydes in excellent enantioselectivities (≥94% *ee*) (Table 6). α-Branched aliphatic aldehydes generally give mixture of products derived from both N and O attack (Section 3.1.4). Cyclohexanone and derivatives were also exclusively aminoxylated (O:N > 100:1) under catalysis by L-proline (1) [65,66,67,68] or its tetrazolyl derivative (2) [69] in excellent enantioselectivities (generally ≥99%) (Figure 16). Bis-aminoxylation may take place and could be suppressed by using large excess of the ketone (10 equiv) and slow addition of nitrosobenzene by a syringe pump. Racemic 3-substituted cyclohexanones and 4-substituted cyclohexanone derivatives give mixture of regio- (3-substituted cyclohexanones only) and diastereomeric aminoxylated products in high enantioselectivities [66,68]. 

**Table 6:** Substrate scopes for L-proline-catalyzed α-aminoxylation of aldehydes with nitrosobenzene. 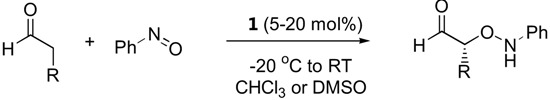

R	conditions	yield (%)^a^	*ee* (%)^a^	ref.
Me, Et, *^n^*Pr, ^i^Pr, Ph, Bn	30 mol% **1**, MeCN, -20 ºC, 24 h	62–>99	95–99	[62]
Me, *^n^*Bu, ^i^Pr, allyl, Bn, Ph, TIPSO(CH_2_)_3_, *N*-methylindol-3-ylmethyl	5 mol% **1**, CHCl_3_, 4 ºC, 4 h	60–88	97–99	[63]
Me, *^n^*Pr, ^i^Pr, *^n^*Bu, allyl, Bn, BnOCH_2_, BocNH(CH_2_)_4_	20 mol% **1**, DMSO, RT, 10–20 min	54–86	94–99	[64]

^a ^Yield and *ee* were determined after NaBH_4_ reduction to the corresponding diols.

**Figure 17:**
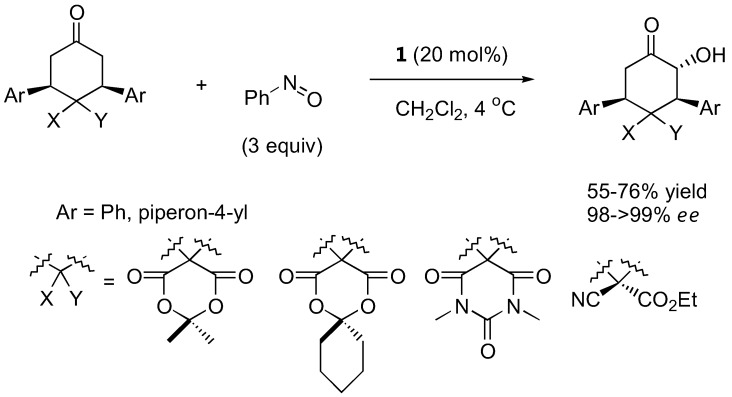
L-Proline (**1**)-catalyzed α-aminoxylation of spirocyclic ketones followed by concomitant N-O bond heterolysis [70].

High enantio- and diastereo-selectivities were also observed with *meso*-3,5-disubstituted spirocyclic ketones [70,71] (Figure 17). Simple acyclic ketones are more problematic substrates as they are prone to multiple aminoxylation and provide appreciable amounts of the hydroxyaminated product (O:N = 78:22–98:2, Section 3.1.4) [65,67,69]. Nevertheless, the aminoxylated products could be obtained in acceptable yield with high enantioselectivity (>99% *ee*) (Figure 16). The reaction took place regioselectively at the more substituted α-carbon of unsymmetrical ketones.

#### 3.1.2. Catalysts

L-Proline (**1**) and its tetrazole analogue (**2**) were the two most widely used catalysts for α-aminoxylation. They are equally effective for simple aldehyde and ketone substrates both in terms of yields and enantioselectivities (usually >95%), although the catalyst loading required to achieve satisfactory results was lower for the latter (5–10 mol%) than the former (10–30 mol%) [69]. Variants to facilitate product isolation including polymer-supported proline catalyst [72]. Ionic liquid as the reaction medium had also been described [73,74]. 

**Figure 18:**
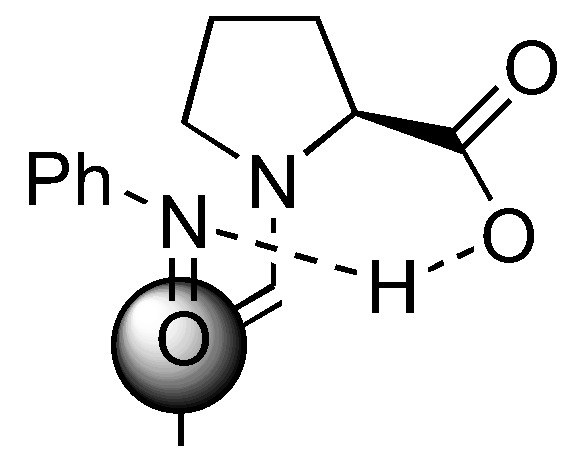
A transition structure involving reaction of proline enamine with nitrosobenzene according to Houk [75] and Córdova [67].

Several models have been proposed to explain the regio- and stereoselectivity of proline catalyzed aminoxylation of aldehydes [62,63,64]. Detailed calculations by Houk [75] and by Córdova [67] suggested that the nitrosobenzene was protonated at the nitrogen atom by the carboxyl group and approached the (*E*)-*anti*-enamine intermediate from the *Re*-face (Figure 18). Houk also predicted that catalysts without hydrogen bonding capability should favor hydroxyamination [75] (Section 3.1.4). 

**Figure 19:**
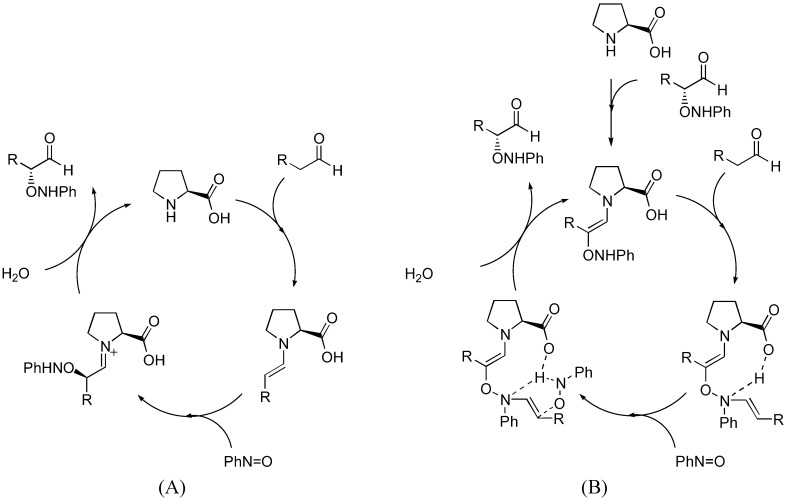
(A) The generally accepted catalytic cycle of proline-catalyzed aminoxylation of aldehydes; (B) The proposed autocatalytic model by Blackmond *et al*. [76].

**Figure 20:**
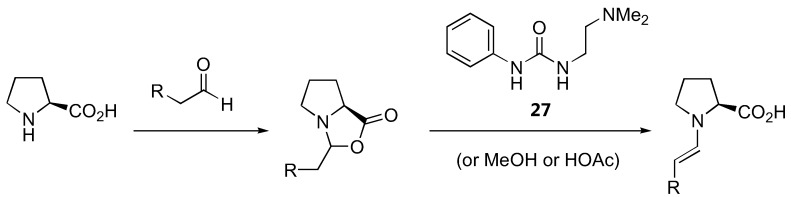
The involvement of urea **27 **in accelerating the rate of proline-catalyzed aminoxylation [77].

The generally accepted catalytic cycle [Figure 19 (A)] involving such enamine intermediate may be somewhat oversimplified. A detailed study of the reaction kinetics of proline-catalyzed aminoxylation of aldehydes by the Blackmond group revealed that the reaction is autocatalytic [76]. In addition to the rate enhancement, the enantiomeric excess gets improved during the course of the reaction [76]. A catalytic cycle involving an adduct of proline with the reaction product was proposed [Figure 19 (B)] to account for this unusual reaction kinetics not observed in other proline-catalyzed reactions except for α-amination [32,33] (see also Section 2.1.1). Addition of a bifunctional urea (**27**) was found to accelerate the rate of proline-catalyzed aminoxylation in nonpolar solvents, but in this case the reaction was no longer autocatalytic [77]. It was suggested that the urea additive might increase the reaction rate by promoting the formation of the enamine from proline *via* the oxazolidinone, which was believed to be the rate limiting step (Figure 20). It was subsequently observed that simple alcohols and acids such as methanol and acetic acid could also increase the reaction rate by promoting the enamine formation [78]. Such additive effect was also observed in the mechanistically related amination but not aldol reactions. The difference in kinetic behavior of these reactions was rationalized by proposing a different rate determining step for each reaction (enamine formation for amination and aminoxylation *vs.* electrophilic reaction of the enamine for aldol reaction) [78]. 

**Table 7:** Summary of performance of other proline- and nonproline-based catalysts in α-aminoxylation of carbonyl compounds. 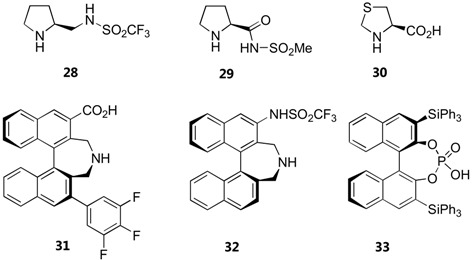

Cat.	Substrates ^a^	Conditions	Yield (%)	*ee* (%)	Ref.
**5**	ketones (7)	10 mol% cat., DMF,	45–76	98–>99	[79]
	α-unbranched aldehydes (2)	0 ºC, a few hours			
**28**	ketones (4)	20 mol% cat., DMSO, RT	66–94	97–>99	[80]
	α-unbranched aldehydes (5)				
**29**	cyclic ketones (4)	10 mol% cat., DMSO, RT	23–80	96–>99	[82]
	α-unbranched aldehydes (1)				
**30**	α-unbranched	20 mol% cat., H_2_O,	74–88	93–>99	[81]
	aldehydes (11)	2 equiv Bu_4_NBr, 0 ºC–> RT			
**31**	α-unbranched aldehydes (3)	5 mol% cat., toluene, 0 ºC	69–89	86–88	[83]
**32**	α-unbranched aldehydes (7)	0.2–5 mol% cat., CHCl_3_,	86­–96	97–>98	[84]
		0 ºC ­–> RT			
**33**	cyclic 1,3-diketones (5)	1 mol% cat., benzene, 4 ºC	49–88	68–>98	[86]
	cyclic β-ketoesters (11)				

^a^ Number of examples shown in parentheses.

In addition to **1** and **2**, other alternative catalysts for direct aminoxylation of aldehydes and ketones include 4-silyloxyproline (**5**) [79], pyrrolidine sulfonamide **28** [80], proline *N*-sulfonyl carboxamide **29** [82] and L-4-thiazolidinecarboxylic acid (L-thiaproline) **30** [81]. The latter was successfully employed for aminoxylation of aldehydes in aqueous media in the presence of Bu_4_NBr as a phase transfer catalyst. Yield and enantioselectivities in most cases are similar to those of unmodified proline, although the silyloxyproline **5** generally requires significantly shorter time and can be used with certain substrates that give poor results with proline such as cycloheptanone [79]. A summary of substrate scope and performance of these catalysts is shown in Table 7.

**Figure 21:**
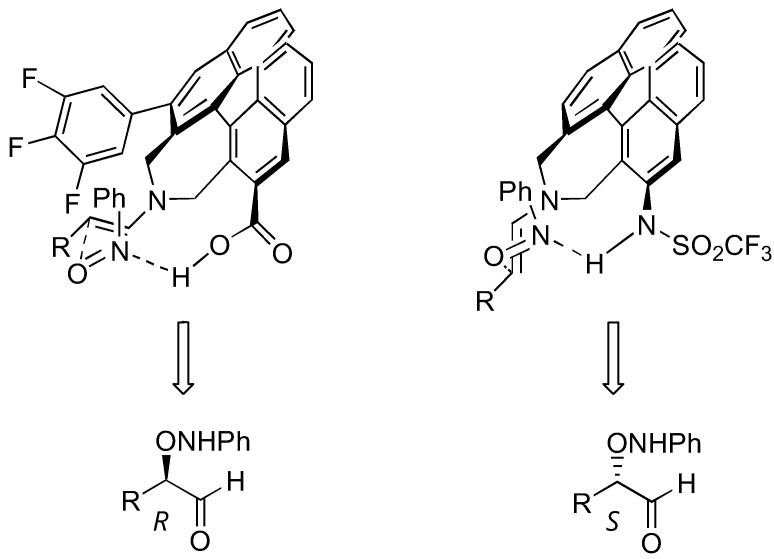
Transition state models to explain the opposite enantioselectivities observed with the catalysts **31** (left) and **32** (right) [85].

A few non-proline derivatives have been reported as effective organocatalysts for aminoxylation reactions. These include Maruoka's axially chiral secondary amino acid **31** [83] and triflamide **32** [84] (Table 7). The triflamide displayed a remarkable catalytic activity. As high as 76% yield and 98% *ee* could be obtained from α-aminoxylation of simple aldehydes at 0.2 mol% catalyst loading. The two catalysts, although having the same axial chirality, gave the aminoxylated products with opposite enantioselectivities. This is explained by the approach of the nitrosobenzene from the *Re* face of the s-*trans*-enamine (catalyst **31**) or the *Si* face of the s-*cis*-enamine (catalyst **32**) (Figure 21) [85]. 

The chiral Brønsted binaphthylphosphoric acid catalyst **33** described by Zhong *et al.* catalyzes α-aminoxylation of cyclic β-dicarbonyl compounds in good yield and excellent regio- and enantioselectivity [86]. Interestingly, the N-O bond heterolysis took place to give the α-hydroxy-β-dicarbonyl compounds without requiring further chemical reduction.

#### 3.1.3. Applications

**Figure 22:**
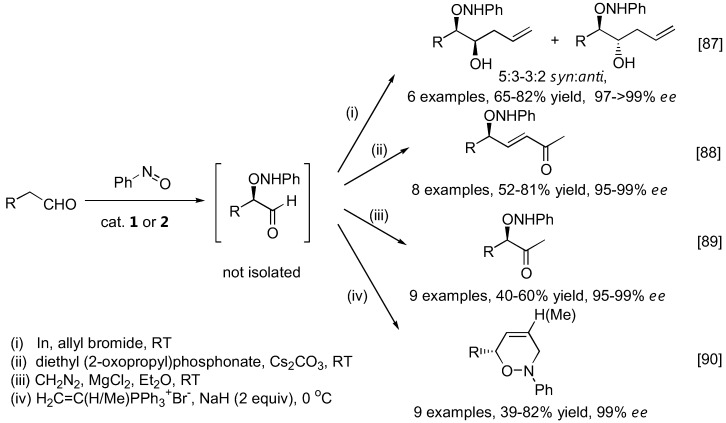
Transformation of aminoxylated aldehydes to other synthetically useful intermediates.

The α-aminoxylation products were generally isolated and characterized following NaBH_4_ reduction of the aldehyde group to give the more stable alcohol. Alternatively, the aminoxylated aldehydes could be transformed into other synthetically useful intermediates (Figure 22) such as substituted homoallylic alcohols [87], allylic alcohols [88] and methyl ketones [89]. In a more complex example, dihydro-1,2-oxazines were obtained from the aminoxylated aldehydes in a one-pot fashion by *N*-alkylation followed by an intramolecular Wittig-type reaction [90]. A similar transformation was also possible with cyclic ketones [91,92]. 

**Figure 23:**
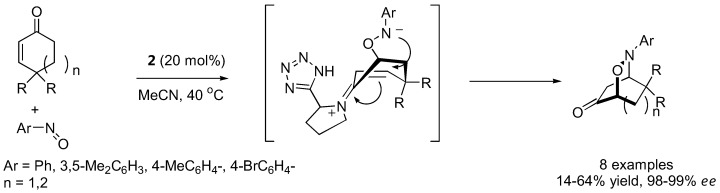
Tandem aminoxylation-intramolecular Michael addition of aminoxylated products derived from unsaturated aldehydes [93,94].

**Figure 24:**
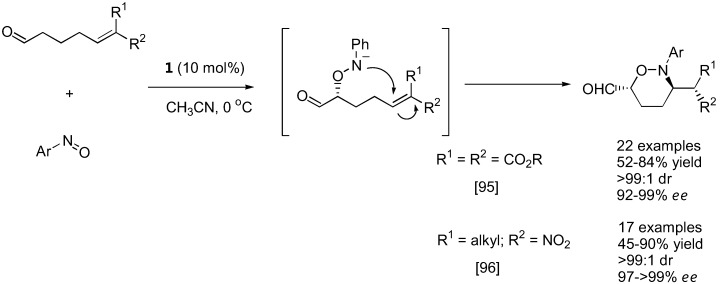
Tandem aminoxylation-intramolecular Michael addition of aminoxylated products derived from unsaturated aldehydes [95,96].

With strategically designed substrates, subsequent reactions may take place spontaneously following the initial aminoxylation. Tetrahydro-1,2-oxazines could be generated from cyclic enones [79,93,94] or 5-hexen-1-al derivatives carrying strong electron withdrawing groups at the 6 position [95,96] by intramolecular Michael-type addition (Figure 23 and Figure 24). An interesting dichotomy was observed in similar aldehyde substrates carrying just one carboxyalkyl instead of the nitro or malonate groups. In these cases, an intramolecular [3+2] cycloaddition between the *in situ* generated nitrone and the alkene was more favorable giving bicyclic isoxazolidines with up to five contiguous stereogenic centers in a one-pot fashion [97].

**Figure 25:**
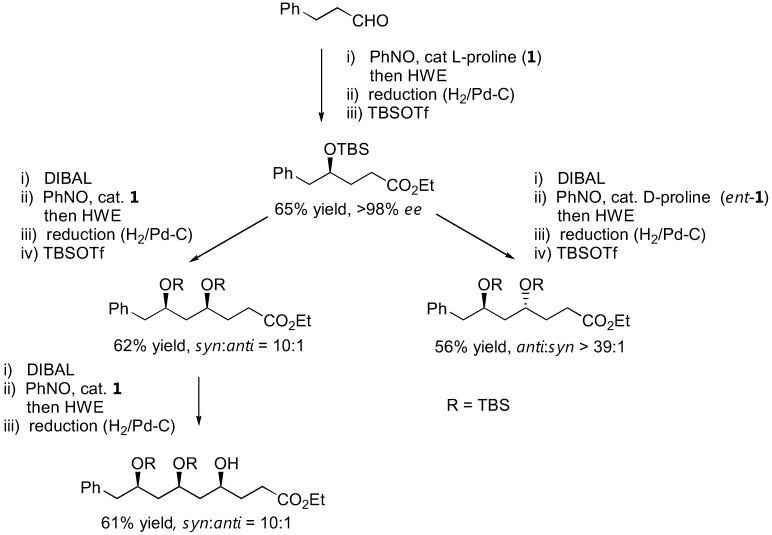
A strategy to synthesize 1,3-polyols by iterative aminoxylation-HWE olefination-reduction sequence [98].

The N-O bond in the aminoxylated carbonyl compounds and their further products can be easily cleaved by a variety of methods, including catalytic amounts of CuSO_4_ in MeOH, Zn/H^+^ reduction or catalytic hydrogenolysis to afford the corresponding 1,2-diols (simple aminoxylated aldehydes), 1,4-aminoalcohols (1,2-oxazine derivatives) [91,92,94], or 1,3-aminoalcohols (bicyclic oxazolidine derivatives) [97] in good enantioselectivities. With excess of the nitrosobenzene in the reaction, N-O heterolysis took place spontaneously [70,86]. It was also possible to apply the aminoxylation-HWE olefination-reduction sequence to construct 1,3-polyol frameworks [98]. The absolute configuration of the newly added hydroxyl group could be reliably controlled by the appropriate choice of L- or D-proline catalyst. Most importantly, the reaction can be carried out in an iterative fashion to give longer 1,3-polyols in a stereocontrolled fashion (Figure 25).

**Figure 26:**
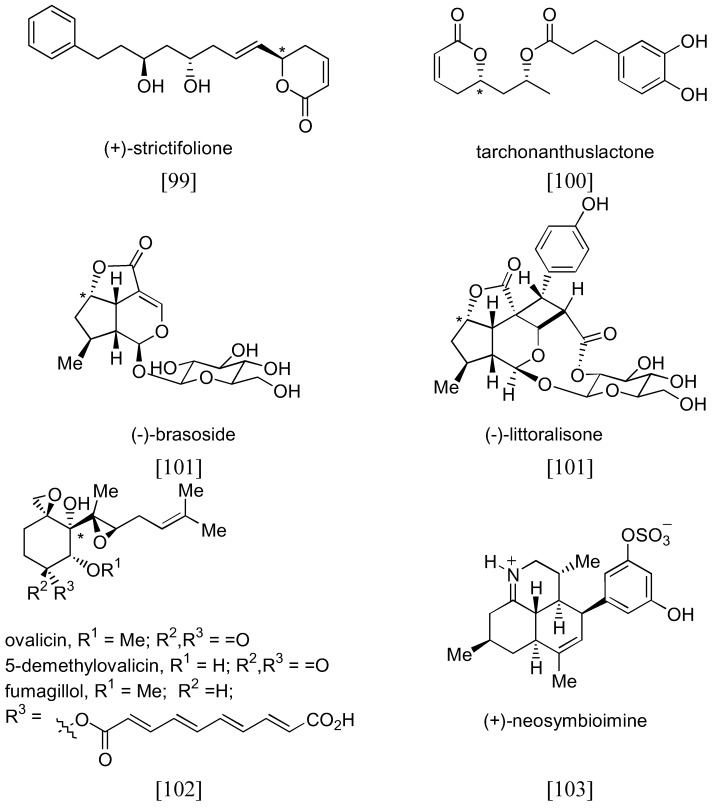
Examples of complex target molecules successfully synthesized by organocatalyzed aminoxylation of aldehydes. The position of the stereogenic center derived from the α-amination is marked by an asterisk.

Numerous applications of organocatalyzed aminoxylation of aldehydes and ketones could be found in the literature [57]. Although a number of new catalysts have been described, the original L- and D-prolines (**1** and *ent*-**1**) were the most widely used catalysts. Examples of complex target molecules successfully synthesized by using this strategy are shown in Figure 26.

Other target molecules which had been successfully synthesized using proline catalyzed α-aminoxylation as one of the key steps include halipeptin A [104], (+)-*exo*- and (–)-*endo*-brevicomins [105], linezolid and eperezolid [106], levetiracetam [107], (*S*,*S*)-ethambutol [108], (+)-harzialactone A and (*R*)-(+)-4-hexanolide [109], (*R*)-seleginine [110], (*S*)-propanolol and (*S*)-naftopidil [111], (+)-disparlure and its *trans*-isomer [112], (-)-anisomycin [59] and (1*R*,3*S*)-thysanone, a HRV 3C-protease inhibitor [113].

#### 3.1.4. α-Aminoxylation *vs.* nitrosoaldol reactions

α-Unbranched aldehydes and cyclic ketones generally react with nitrosobenzene exclusively at the oxygen atom under catalysis by proline (Section 3.1.1). Significant amounts of products derived from the attack at the nitrogen atom were observed with acyclic ketones [67,69] and α-branched aliphatic aldehydes [114] (Table 8, see also Figure 16). 

**Table 8:** Comparison of catalysts in aminoxylation/nitrosoaldol reactions of aldehydes. 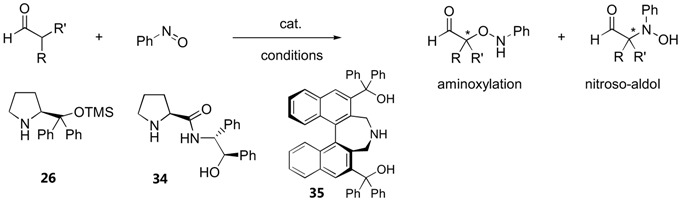

Cat.	Substrate^a^	Conditions	Yield (%)	*ee* (%)^b^	Ref.
**1**	2-methyl-3-propanal	20 mol% cat., DMF,	32	67	[114]
		25 ºC, 24 h	(N:O = 1.5:1)		
**2**	α-branched aldehydes (10)	20 mol% cat., DMF,	55–96	5–90	[114]
		0–25 ºC , 3–24 h	(N:O = 0.6:1–20:1)		
**34**	α-branched aldehydes (5)	10 mol% cat., toluene,	53–74	46–59	[115]
		-40 ºC, 2–3 d	(N:O = N/A)		
**26**	α-unbranched aldehydes (8)	20 mol% cat., CH_2_Cl_2_,	40–75	91–99	[116]
		0 ºC	(N:O >99:1)		
**35**	α-unbranched aldehydes (6)	10 mol% cat., THF,	70–90	96–99	[117]
		0 ºC, 1 h	(N:O >99:1)		

^a^ Number of examples shown in parentheses ^b^*ee* of the *N*-nitrosoaldol product.

Steric effects between the substituent in the enamine intermediate and the phenyl group in nitrosobenzene had been proposed to facilitate the attack at nitrogen [114]. Despite this possibility of direct *N*-nitrosoaldol reaction, the N:O selectivity was not generally synthetically useful (0.6:1–1.7:1), except in two cases where the substrates were 2-phenylpropanal derivatives, whereby a very good N:O selectivity (10:1–20:1) could be obtained. Enantioselectivities for the nitrosoaldol products were only moderate to good (45–90%) for α-methyl substituted aldehydes and poor (5–25%) for the corresponding α-ethyl derivatives [114]. The L-prolinamide derived from aminoalcohol (**34**) was demonstrated to catalyze a selective nitrosoaldol reaction of α-branched aliphatic aldehydes in good yield (53–74%) with moderate *ee* (46–59%) at 10 mol% catalyst loading [115]. Under similar conditions, L-proline (**1**) gave only the aminoxylation product in low *ee* (37%). The preferential hydrogen bonding between the oxygen atom in nitrosobenzene and weakly acidic amide and hydroxyl protons was proposed to account for the high *N*-selectivity. In line with the prediction by Houk [75], the non-hydrogen bonding diphenylprolinol TMS ether (**26**) catalyzed the amination reaction between simple unbranched aliphatic aldehydes and nitrosobenzene to give exclusively the nitrosoaldol product in 40–75% yield with >99:1 N:O selectivity and excellent enantioselectivities (91–99%)[116]. The absolute configuration of the product was consistent with the approach of the nitrosobenzene from the *Si* face of the (*E*)-enamine, which is opposite to that of proline and its derivatives with hydrogen-bonding substituents. A DFT calculation supported the preferential attack on N over O by >3 kcal/mol [116]. 

A few non-proline derivatives have recently been demonstrated as effective catalysts for selective nitrosoaldol reactions of carbonyl compounds and related derivatives. The most general one is perhaps the axially chiral secondary aminoalcohol (**35**) developed by Maruoka *et al.* [117], which provide excellent N:O selectivity (>99:1) and enantioselectivities (≥96%) in nitrosoaldol reaction of simple α-unbranched aldehydes (Table 8). 

**Figure 27:**
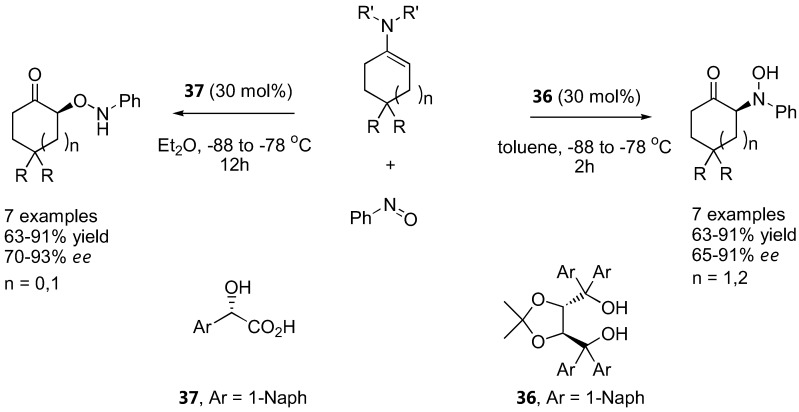
Regioselectivity control in reaction between a preformed enamine and nitrosobenzene by Brønsted acid catalysts [118].

It should be noted that more strongly acidic substituents in place of the alcohol in the structurally related catalysts **31** and **32** provide preferentially *O*-attack products due to protonation of the nitrogen atom (Section 3.1.2) [83,84]. Other catalysts act on special substrates. The reaction between achiral morpholino-enamines derived from cyclohexanone and cycloheptanone derivatives with nitrosobenzene in the presence of a TADDOL derivative (**36**) (30 mol%) provided the nitrosoaldol product in good yields (63–91%) and enantioselectivities (65–91%) [118]. The N:O regioselectivity could be completely reversed using 1-naphthylglycolic acid (**37**) as a more strongly Brønsted acid catalyst. Good yields (63–91%) and enantioselectivities (70–93%) of the *O*-substituted products were also obtained (Figure 27). 

**Figure 28:**
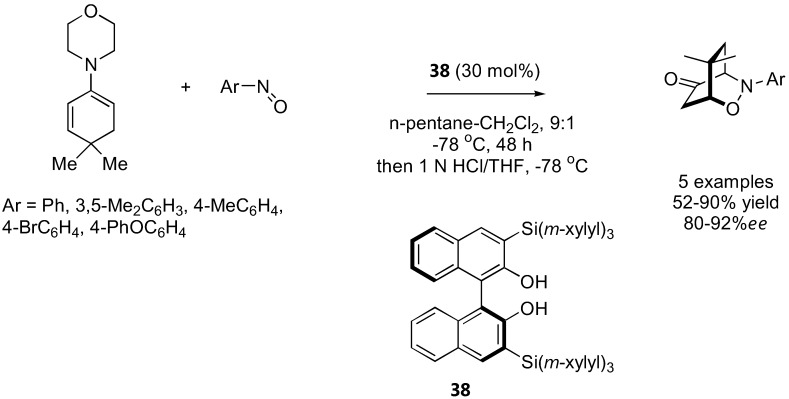
Binaphthol catalyzed tandem hydroxyamination-Michael addition of cyclohexenone enamine [94].

The binaphthol derivative **38** catalyzed regio- and enantio-selective hydroxyamination of cyclohexanone and cyclohexenone enamines [94]. With the cyclohexenone enamine, the reaction was followed by an intramolecular Michael addition to give a bicyclic ketone analogous to those obtained from α-aminoxylation of cyclohexenone with proline catalysis [93] (Section 3.1.3), but with complete reversal of regioselectivity (Figure 28). 

**Figure 29:**
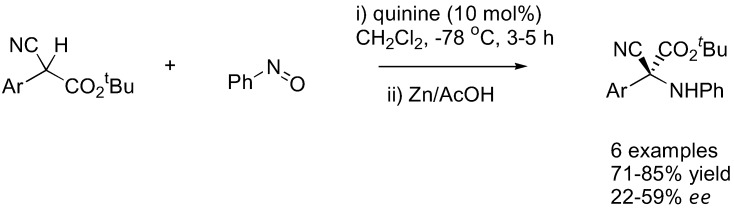
Nitrosoaldol reaction of α-aryl substituted cyanoacetate esters [119].

Under catalysis by quinine (**11**), α-aryl substituted cyanoacetate esters underwent exclusive nitrosoaldol reactions to give the chiral quaternary center in good yield (71–85%) and moderate *ee* (22–59%) (Figure 29) [119]. The enantioselectivity of the reaction is highly dependent to the solvent and concentration of the catalyst, which was explained by the different aggregation state of the catalyst.

### 3.2. α-Aminoxylation reactions with TEMPO

**Figure 30:**
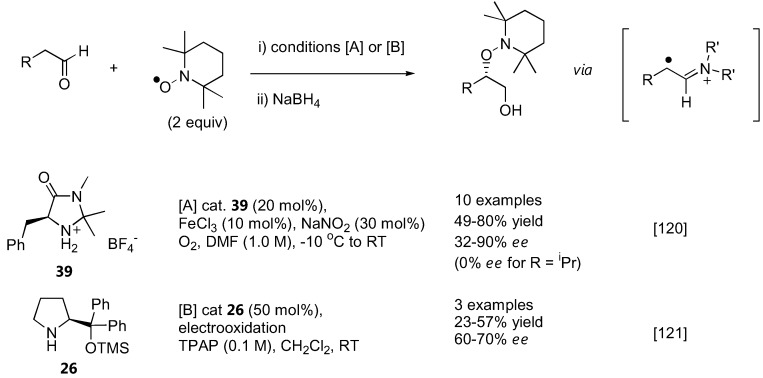
α-Aminoxylation reactions *via* radical intermediates with TEMPO [120,121].

While all aminoxylation reactions described above used nitrosobenzene or substituted nitrosobenzenes as the aminoxylating agents, Sibi and Hasegawa have recently introduced a conceptually new approach to aminoxylate aldehydes using the stable radical TEMPO [120] (Figure 30). In this case, a radical intermediate was generated from a chiral secondary amine-derived enamine by an appropriate single electron transfer (SET) reagent. This radical can then be trapped with TEMPO, resulting in an overall α-aminoxylation of the carbonyl compound. A combination of FeCl_3_ (10 mol%) and NaNO_2_ (30 mol%) was found to be the best SET reagent. A MacMillan's imidazolinone catalyst **39** was more effective than proline and diphenylprolinol both in terms of yield and enantioselectivities. Only unbranched aldehydes with terminal π-system within 2–3 atoms from the reaction center such as 2-phenylpropanal and 4-penten-1-al gave optimal enantioselectivities (up to 90% *ee*) at 20 mol% catalyst loading. Simple aliphatic aldehydes such as isovaleraldehyde gave the product in 74% yield, but without enantioselectivity (0% *ee*). The chiral enamine radicals derived from **26** could also be generated by anodic oxidation, and subsequently trapped by TEMPO to give the aminoxylated aldehydes [121]. The yield and enantioselectivity was not high, but the substrates chosen in the latter [121] also gave poor results in the former reports [120].

## 4. α-Oxidation Reactions

### 4.1. α-Oxybenzoylation

**Figure 31:**
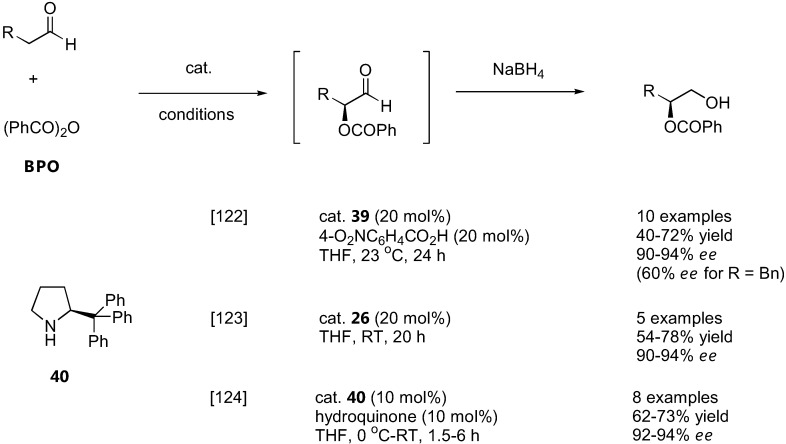
Organocatalyzed α-oxybenzoylation of aldehydes with benzoyl peroxide.

Three direct organocatalytic asymmetric α-oxybenzoylations of carbonyl compounds by benzoyl peroxide (BPO) have been independently reported in early 2009 by Tomkinson [122], Hayashi [123] and Maruoka [124] (Figure 31). Hayashi's group showed that the diphenylprolinol silyl ether **26** was an effective catalyst for this reaction [123]. 

On the other hand, the Tomkinson's group employed a MacMillan's imidazolinone catalyst (**39**) and the Maruoka's group used a tritylpyrrolidine catalyst (**40**), both of which were thought to be more stable to oxidation by BPO than simple proline derivatives [122,124]. In all cases, similar substrate scopes (simple α-unbranched aliphatic aldehydes, some carrying inert functional groups such as aromatic, alkenyl, ether or phthalimido), yield (40–78%) and *ee* (90–94%) were reported. The stereochemical outcome of the reaction was consistent with the simple electrophilic attack of the *E*-enamine intermediate from the less hindered face. The resulting α-oxybenzoylated aldehydes were successfully transformed into a variety of products by standard chemical manipulation of the aldehyde group [122,124].

### 4.2. α-Oxidation with molecular oxygen

**Figure 32:**
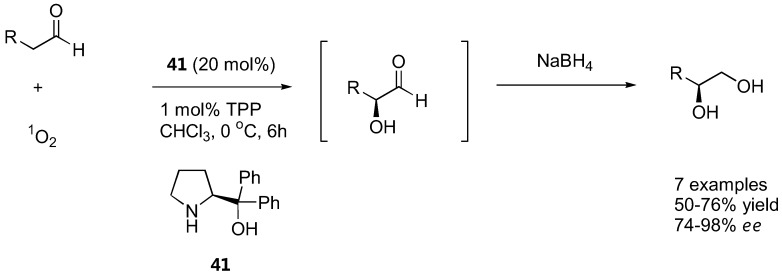
Organocatalyzed α-oxidation with molecular oxygen [125].

Following their 2004 report of L-α-methylproline-catalyzed oxidation of aldehydes by singlet oxygen to give directly the optically active hydroxyaldehydes and diols (after reduction) in up to 64% *ee* [126], the Córdova group has again shown in 2006 that diphenylprolinol (**41**) was a more effective for direct α-oxidation of α-unbranched aldehydes with singlet oxygen [125] (Figure 32). The singlet oxygen was photochemically generated from oxygen or air in the presence of a catalytic amount of tetraphenylphorphine (TPP). The reaction was carried out in the presence of the diphenylprolinol catalyst (10–20 mol%) at 0 ºC. The intermediate α-hydroperoxyaldehydes was not isolated, but further reduced by NaBH_4_ to afford the corresponding diols in moderate to good yield (50–76%). The *ee* was moderate with simple aliphatic aldehydes (propanal: 74% *ee*, 1-hexanal: 75% *ee*) but was dramatically improved with 2-phenylpropanal and its derivatives (up to 98% *ee*).

### 4.3. α-Oxidation with hydroperoxides

**Figure 33:**
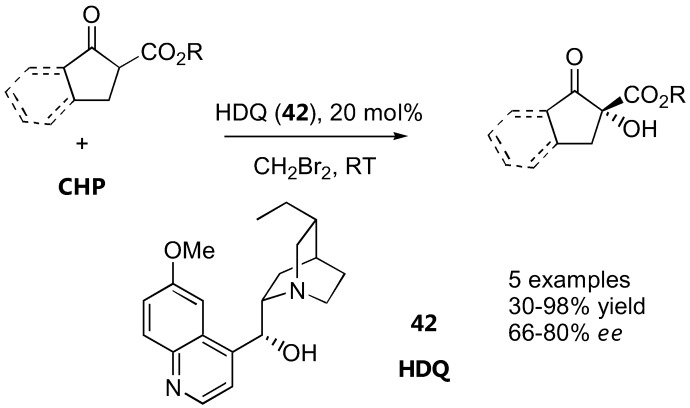
Organocatalyzed α-oxidation of cyclic β-ketoesters with hydroperoxides [127].

Alkyl cyclopentanone 2-carboxylate and its benzo-derivatives were oxidized with cumyl hydroperoxide (CHP) in the presence of catalytic amounts of dihydroquinine (HDQ, **42**) to give α-hydroxy-β-keto esters in 66–80% *ee* (Figure 33). Subsequent borane reduction gave the *anti*-diol in an excellent diastereoselectivity (99:1) [127]. 

**Figure 34:**
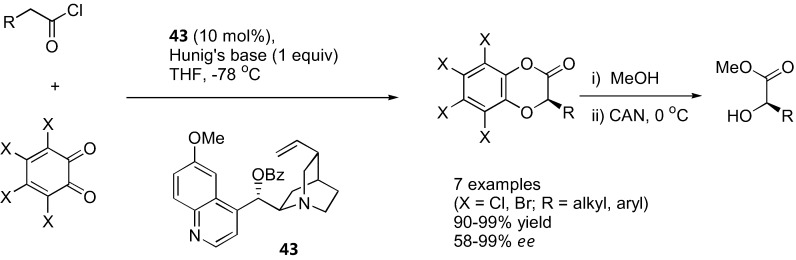
Organocatalyzed α-aryloxylation of carboxylic acid derivatives *via* ketene enolates [128].

**Figure 35:**
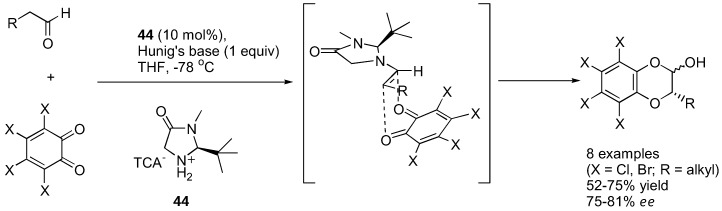
Organocatalyzed α-aryloxylation of aldehydes by *o*-quinones [129].

### 4.4. α-Aryloxylation with o-quinones

Lectka's group showed that *o*-chloranil and *o*-bromonil can react enantioselectively with ketene enolates in a [4+2] cycloaddition fashion to give 1,4-benzodioxane-2-one under catalysis by a cinchona alkaloid derivative **43** [128] (Figure 34). The ketene enolates were generated from their corresponding acid chloride in the presence of Hünig's base. Excellent yields and good enantioselectivities were obtained in most cases. The cycloadducts could be converted into the corresponding α-hydroxy esters by methanolysis followed by ceric ammonium nitrate (CAN) treatment. Similarly, α-unbranched aliphatic aldehydes reacted with *o*-chloranil or *o*-bromonil under organocatalysis to give the α-aryloxylated aldehydes in their hemiacetal form [129] (Figure 35). The MacMillan type catalyst (**44**) provided superior results in aryloxylation of propionaldehyde (>70% yield and >80% *ee*) than proline and its simple derivatives (36–53% yield, 33–40% *ee*). A range of α-unbranched aliphatic aldehydes were acceptable substrates, providing *ee* in the range of 75–81%. The hemiacetal products were successfully converted to alcohols and 1,4-benzodioxanes without loss of optical purities.

### 4.5. α-Oxidation with oxaziridine and iodosobenzene

**Figure 36:**
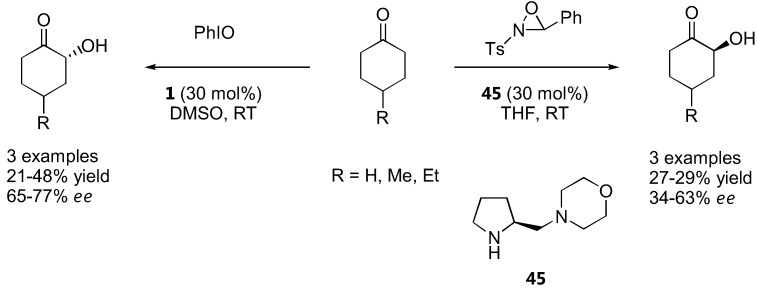
Proline and diamine **45** catalyzed α-oxidation of ketones with oxaziridine and iodosobenzene [130].

Cyclohexanones could be oxidized by an *N*-sulfonyloxaziridine in the presence of proline (**1**) or its derivatives to the corresponding α-hydroxyketones in poor to moderate yield and enantioselectivity. The best result was obtained with the diamine **45** (up to 63% *ee*) [130] (Figure 36). 

**Figure 37:**
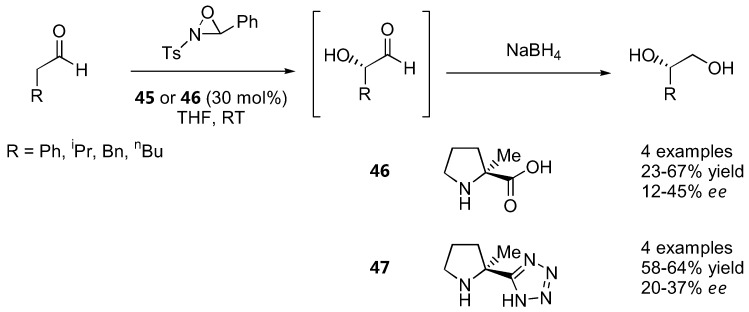
α-Methylproline (**46**) and tetrazole (**47**) catalyzed α-oxidation of aldehydes with oxaziridine [131].

Iodosobenzene was a superior oxidant for cyclohexanone derivatives with unmodified L-proline (**1**) as catalyst and *ee* in the range of 65–77% were obtained [130] (Figure 36). The absolute configurations of the oxidation products derived from proline-catalyzed reactions were opposite to that of the diamine-catalyzed reactions regardless of the oxidant used, which could be explained by their different transition states (see 2.1.1). Simple α-unbranched aldehydes could also be oxidized to the corresponding α-hydroxyaldehydes by *N*-sulfonyloxaziridine with α-methylproline (**46**) or its tetrazole analogue **47** as catalysts. Interestingly, the (*S*)-product predominated instead of the expected (*R*)-product in analogy to the amination and aminoxylation reactions, but the *ee* was rather poor (<50%) [131] (Figure 37).

### 4.6. α-Oxysulfonation catalyzed by iodoarenes

**Figure 38:**
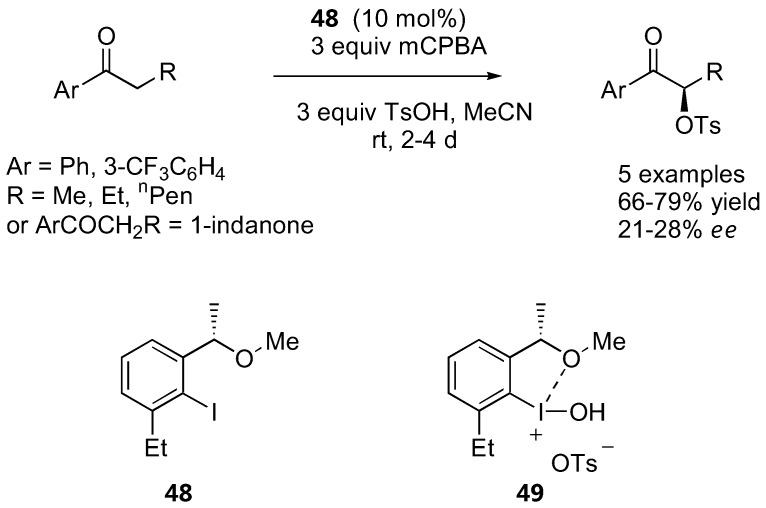
α-Oxysulfonation of aromatic ketones catalyzed by iodoarenes [132,133].

While aryl ketones are generally unreactive towards enamine catalysis by proline derivatives (see also Section 2.1.2), propiophenone derivatives have been successfully oxidized to the corresponding α-tosyloxy derivatives by chiral Koser-type reagents catalytically generated *in situ* from the corresponding iodoarenes, *m*-CPBA and *p*-toluenesulfonic acid at room temperature [132,133] (Figure 38).

Extensive screening of the iodoarenes revealed that the best catalyst precursor was **48**, which was presumably converted into the active oxidizing agent **49***in situ*. Although conceptually unique, the *ee* was not particularly good (21–28%) and no improvement could be made by changing the steric bulk of the sulfonic acids used [133].

## 5. Conclusions and Outlook

During the past few years an exciting advancement in the field of organocatalyzed asymmetric α-amination, α-aminoxylation, and related α-oxidation process has been witnessed. With high and predictable stereoselectivity, operational simplicity, and catalyst availability in both enantiomers with a touch of "green" aspect, the proline catalyzed α-aminoxylation and α-amination of carbonyl compounds have become a well established tool for constructing complex chiral molecules. Nevertheless, certain limitations still exist with respect to the low catalyst turnover numbers and frequency, which results in a typically high catalyst loading (≥10 mol%) and long reaction times (several hours to several days). These limitations, together with the rather limited substrate scope, prompted the design of new alternative catalysts that are more active, provide higher selectivity, and accept a broader substrate range. It is also important to find new catalysts that can catalyze new reactions, or can accept certain "difficult" substrates that proline cannot – most notably α-branched or aromatic substrates. Many of such new catalysts, such as those derived from cinchona alkaloids or other chiral amines as the source of chirality, are presently discovered by trial-and-error. With an emerging understanding as to how the catalysts work, it will clearly lead to a more rational design of new catalysts with such properties in the future. Finally, creative combination of new catalysts and new reaction sequences will further broaden the scope and applicability of these organocatalyzed reactions. It remains to be seen when it will be possible to design an organocatalyst that can catalyze a specific reaction or reaction sequence with enzyme-like efficiency at will. By that time, the traditional use of highly reactive and environmentally unfriendly metal-based reagents and catalysts may become obsolete! 

## References

[1] Merino P., Tejero T. (2004). Organocatalyzed asymmetric α-aminoxylation of aldehydes and ketones - An efficient access to enantiomerically pure α-hydroxycarbonyl compounds, diols, and even amino alcohols. Angew. Chem. Int. Ed..

[2] Janey J.M. (2005). Recent advances in catalytic, enantioselective α aminations and α oxygenations of carbonyl compounds. Angew. Chem. Int. Ed..

[3] Marigo M., Jørgensen K.A. (2006). Organocatalytic direct asymmetric α-heteroatom functionalization of aldehydes and ketones. Chem. Commun..

[4] Guillena G., Ramón D.J. (2006). Enantioselective α-heterofunctionalisation of carbonyl compounds: organocatalysis is the simplest approach. Tetrahedron-Asymmetr.

[5] Marigo M., Jørgensen K.A., Dalko P.I. (2007). Enamine Catalysis. Enantioselective Organocatalysis: Reactions and Experimental Procedures.

[6] Kotsuki H., Ikishima H., Okuyama A. (2008). Organocatalytic asymmetric synthesis using proline and related molecules. Part 2. Heterocycles.

[7] Kano T., Maruoka K. (2008). Design of chiral bifunctional secondary amine catalysts for asymmetric enamine catalysis. Chem. Commun..

[8] Chen Y.-C. (2008). The development of asymmetric primary amine catalysts based on cinchona alkaloids. Synlett.

[9] Yamamoto H., Momiyama N. (2005). Rich chemistry of nitroso compounds. Chem. Commun..

[10] Yamamoto H., Kawasaki M. (2007). Nitroso and azo compounds in modern organic synthesis: Late blooming but very rich. Bull. Chem. Soc. Jpn..

[11] Enders D., Grondal C., Hüttl R.M. (2007). Asymmetric organocatalytic domino reactions. Angew. Chem. Int. Ed..

[12] Bøgevig A., Juhl K., Kumaragurubaran N., Zhuang W., Jørgensen K.A. (2002). Direct organo-catalytic asymmetric α-amination of aldehydes–A simple approach to optically active α-amino aldehydes, α-amino alcohols, and α-amino acids. Angew. Chem. Int. Ed..

[13] List B. (2002). Direct catalytic asymmetric α-amination of aldehydes. J. Am. Chem. Soc..

[14] Jung S.H., Kim D.Y. (2008). Catalytic enantioselective electrophilic α-hydrazination of β-ketoesters using bifunctional organocatalysts. Tetrahedron Lett..

[15] Terada M., Nakano M., Ube H. (2006). Axially chiral guanidine as highly active and enantioselective catalyst for electrophilic amination of unsymmetrically substituted 1,3-dicarbonyl compounds. J. Am. Chem. Soc..

[16] He R., Wang X., Hashimoto T., Maruoka K. (2008). Binaphthyl-modified quaternary phosphonium salts as chiral phase-transfer catalysts: Asymmetric amination of β-keto esters. Angew. Chem. Int. Ed..

[17] Xu X., Yabuta T., Yuan P., Takemoto Y. (2006). Organocatalytic enantioselective hydrazination of 1,3-dicarbonyl compounds: Asymmetric synthesis of α,α-disubstituted α-amino acids. Synlett.

[18] Dahlin N., Bøgevig A., Adolfsson H. (2004). *N*-Arenesulfonyl-2-aminomethylpyrrolidines - Novel modular ligands and organocatalysts for asymmetric catalysis. Adv. Synth. Catal..

[19] Franzén J., Marigo M., Fielenbach D., Wabnitz T., Kjærsgaard A., Jørgensen K.A. (2005). A general organocatalyst for direct α-functionalization of aldehydes: Stereoselective C-C, C-N, C-F, C-Br, and C-S bond-forming reactions. Scope and mechanistic insights. J. Am. Chem. Soc..

[20] Nishikawa Y., Kitajima M., Takayama H. (2008). First asymmetric total syntheses of cernuane-type Lycopodium alkaloids, cernuine, and cermizine D. Org. Lett..

[21] Vogt H., Vanderheiden S., Bräse S. (2003). Proline-catalysed asymmetric amination of α,α-disubstituted aldehydes: synthesis of configurationally stable enantioenriched α-aminoaldehydes. Chem. Commun..

[22] Baumann T., Vogt H., Bräse S. (2007). The proline-catalyzed asymmetric amination of branched aldehydes. Eur. J. Org. Chem..

[23] Chowdari N.S., Barbas C.F. (2005). III, Total synthesis of LFA-1 antagonist BIRT-377 *via* organocatalytic asymmetric construction of a quaternary stereocenter. Org. Lett..

[24] Baumann T., Bächle M., Hartmann C., Bräse S. (2008). Thermal effects in the organocatalytic asymmetric α-amination of disubstituted aldehydes with azodicarboxylates: A high-temperature organocatalysis. Eur. J. Org. Chem..

[25] Suri J.T., Steiner D., Barbas C.F. (2005). III Organocatalytic enantioselective synthesis of metabotropic glutamate receptor ligands. Org. Lett..

[26] Kumaragurubaran N., Juhl K., Zhuang W., Bøgevig A., Jørgensen K.A. (2002). Direct L-proline-catalyzed asymmetric α-amination of ketones. J. Am. Chem. Soc..

[27] Thomassigny C., Prim D., Greck C. (2006). Amino acid-catalyzed asymmetric α-amination of carbonyls. Tetrahedron Lett..

[28] Lacoste E., Vaique E., Berlande M., Pianet I., Vincent J.-M., Landais Y. (2007). Benzimidazole-pyrrolidine/H^+^ (BIP/H^+^), a highly reactive organocatalyst for asymmetric processes. Eur. J. Org. Chem..

[29] Hayashi Y., Aratake S., Imai Y., Hibino K., Chen Q.-Y., Yamaguchi J., Uchimaru T. (2008). Direct asymmetric α-amination of cyclic ketones catalyzed by siloxyproline. Chem. Asian J..

[30] Kotrusz P., Alemayehu S., Toma Š., Schmalz H.-G., Adler A. (2005). Enantioselective organocatalysis in ionic liquids: Addition of aliphatic aldehydes and ketones to diethyl azodicarboxylate. Eur. J. Org. Chem..

[31] Dinér P., Kjærsgaard A., Lie M.A., Jørgensen K.A. (2008). On the origin of the stereoselectivity in organocatalysed reactions with trimethylsilyl-protected diarylprolinol. Chem. Eur. J..

[32] Iwamura H., Methew H.P., Blackmond D.G. (2004). *In situ* catalyst improvement in the proline-mediated α-amination of aldehydes. J. Am. Chem. Soc..

[33] Iwamura H., Wells D.H., Mathew S.P., Klussmann M., Armstrong A., Blackmond D.G. (2004). Probing the active catalyst in product-accelerated proline-mediated reactions. J. Am. Chem. Soc..

[34] Mathew S.P., Klussmann M., Iwamara H., Wells D.H., Armstrong A., Blackmond D.G. (2006). A mechanistic rationalization of unusual kinetic behavior in proline-mediated C-O and C-N bond-forming reactions. Chem. Commun..

[35] Liu T.-Y., Cui H.-L., Zhang Y., Jiang K., Du W., He Z.-Q., Chen Y.C. (2007). Organocatalytic and highly enantioselective direct α-amination of aromatic ketones. Org. Lett..

[36] Pihko P.M., Pohjakallio A. (2004). Enantioselective organocatalytic Diels aminations: α-Aminations of cyclic β-keto esters and β-keto lactones with cinchonidine and cinchonine. Synlett.

[37] Saaby S., Bella M., Jørgensen K.A. (2004). Asymmetric construction of quaternary stereocenters by direct organocatalytic amination of α-substituted α-cyanoacetates and β-dicarbonyl compounds. J. Am. Chem. Soc..

[38] Liu X., Sun B., Deng L. (2009). Catalytic enantioselective electrophilic aminations of acyclic α-alkyl β-carbonyl nucleophiles. Synlett.

[39] Zhu R., Zhang D., Wu J., Liu C. (2007). Theoretical study on the enantioselective α-amination reaction of 1,3-dicarbonyl compounds catalyzed by a bifunctional-urea. Tetrahedron Asymmetry.

[40] Liu X., Li H., Deng L. (2005). Highly enantioselective amination of α-substituted α-cyanoacetates with chiral catalysts accessible from both quinine and quinidine. Org. Lett..

[41] Liu Y., Melgar-Fernández Juaristi (2007). Enantioselective amination of α-phenyl-α-cyanoacetate catalyzed by chiral amines incorporating the α-phenylethyl auxiliary. Eur. J. Org. Chem..

[42] Melgar-Fernández R., González-Olvera R., Olivares-Romero J.L., González-López V., Romero-Ponce L., Ramírez-Zárate M.R., Demare P., Regla I., Juaristi E. (2008). Synthesis of novel derivatives of (1*S*,4*S*)-2,5-diazabicyclo[2.2.1]heptane and their evaluation as potential ligands in asymmetric catalysis. Eur. J. Org. Chem..

[43] Kim S.M., Lee J.H., Kim D.Y. (2008). Enantioselective Direct amination of α-cyanoketones catalyzed by bifunctional organocatalysts. Synlett.

[44] Ait-Youcef R., Sbargoud K., Moreau X., Greck C. (2009). Asymmetric α-amination of chiral protected β-hydroxyaldehydes catalyzed by proline. Synlett.

[45] Ait-Youcef R., Kalch D., Moreau X., Greck C. (2009). Asymmetric α-amination of aldehydes and ketones catalyzed by *tert*-butoxy-L-proline. Lett. Org. Chem..

[46] Cheng L., Liu L., Wang D., Chen Y.-J. (2009). Highly enantioselective and organocatalytic α-amination of 2-oxindoles. Org. Lett..

[47] Bui T., Borregan M., Barbas C.F. (2009). Expanding the scope of cinchona alkaloid-catalyzed enantioselective α-aminations of oxindoles: A versatile approach to optically active 3-amino-2-oxindole derivatives. J. Org. Chem..

[48] Bertelsen S., Marigo M., Brandes S., Dinér P., Jørgensen K.A. (2006). Dienamine catalysis: Organocatalytic asymmetric γ-amination of α,β unsaturated aldehydes. J. Am. Chem. Soc..

[49] Poulsen T.B., Alemparte C., Jørgensen K.A. (2005). Enantioselective organocatalytic allylic amination. J. Am. Chem. Soc..

[50] Chowdari N.S., Ramachary D.B., Barbas III C.F. (2003). Organocatalytic asymmetric assembly reactions: One-pot synthesis of functionalized β-amino alcohols from aldehydes, ketones, and azodicarboxylates. Org. Lett..

[51] Umbreen S., Brockhaus M., Ehrenberg H., Schmidt B. (2006). Norstatines from aldehydes by sequential organocatalytic α-amination and Passerini reaction. Eur. J. Org. Chem..

[52] Oelke A.J., Kumarn S., Longbottom D.A., Ley S.V. (2006). An enantioselective organocatalytic route to chiral 3,6-dihydropyridazines from aldehydes. Synlett.

[53] Kotkar S.P., Chavan V.B., Sudalai A. (2007). Organocatalytic sequential α-amination-Horner-Wadsworth-Emmons olefination of aldehydes: Enantioselective synthesis of γ-amino-α,β-unsaturated esters. Org. Lett..

[54] Lim A., Choi J.H., Tae J. (2008). Organocatalytic α-amination-allylation-RCM strategy: enantioselective synthesis of cyclic hydrazines. Tetrahedron Lett..

[55] Marigo M., Schulte T., Franzén, Jørgensen K.A. (2005). Asymmetric multicomponent domino reactions and highly enantioselective conjugated addition of thiols to α,β-unsaturated aldehydes. J. Am. Chem. Soc..

[56] Jiang H., Nielsen J.B., Nielsen M., Jørgensen K.A. (2007). Organocatalysed asymmetric β-amination and multicomponent *syn*-selective diamination of α,β-unsaturated aldehydes. Chem. Eur. J..

[57] de Figueiredo R.M., Christmann M. (2007). Organocatalytic synthesis of drugs and bioactive natural products. Eur. J. Org. Chem..

[58] Kalch D., De Rycke N., Mareau X., Greck C. (2009). Efficient syntheses of enantioenriched (*R*)-pipecolic acid and (*R*)-proline *via* electrophilic organocatalytic amination. Tetrahedron Lett..

[59] Chouthaiwale P.V., Kotkar S.P., Sudalai A. (2009). Formal synthesis of (-)anisomycin *via* organocatalysis. ARKIVOC.

[60] Hartmann C.E., Gross P.J., Nieger M., Bräse S. (2009). Towards an asymmetric synthesis of the bacterial peptide deformylase (PDF) inhibitor fumimycin. Org. Biomol. Chem..

[61] Vogt H., Baumann R., Nieger M., Bräse S. (2006). Direct asymmetric α-sulfamidation of α-branched aldehydes: A novel approach to enamine catalysis. Eur. J. Org. Chem..

[62] Hayashi Y., Yamaguchi J., Hibino K., Shoji M. (2003). Direct proline catalyzed asymmetric α-aminooxylation of aldehydes. Tetrahedron Lett..

[63] Brown S.P., Brochu M.P., Sinz C.J., MacMillan D.W.C. (2003). The direct and enantioselective organocatalytic α-oxidation of aldehydes. J. Am. Chem. Soc..

[64] Zhong G. (2003). A facile and rapid route to highly enantiopure 1,2-diols by novel catalytic asymmetric α-aminoxylation of aldehydes. Angew. Chem. Int. Ed..

[65] Bøgevig A., Sundén H., Córdova A. (2004). Direct catalytic enantioselective α-aminoxylation of ketones: A stereoselective synthesis of α-hydroxy and α,α'-dihydroxy ketones. Angew. Chem. Int. Ed..

[66] Hayashi Y., Yamaguchi J., Sumiya T., Shoji M. (2004). Direct proline-catalyzed asymmetric α-aminoxylation of ketones. Angew. Chem. Int. Ed..

[67] Córdova A., Sundén H., Bøgevig A., Johannson M., Himo F. (2004). The direct catalytic asymmetric α-aminooxylation reaction: Development of stereoselective routes to 1,2-diols and 1,2-amino alcohols and density functional calculations. Chem. Eur. J..

[68] Hayashi Y., Yamaguchi J., Sumiya T., Hibino K., Shoji M. (2004). Direct proline-catalyzed asymmetric α-aminoxylation of aldehydes and ketones. J. Org. Chem..

[69] Momiyama N., Torii H., Yamamoto H. (2004). *O*-Nitroso aldol synthesis: Catalytic enantioselective route to α-aminooxy carbonyl compounds *via* enamine intermediate. Proc. Natl. Acad. Sci. USA.

[70] Ramachary D.B., Barbas C.F. (2005). III Direct amino acid-catalyzed asymmetric desymmetrization of meso-compounds: Tandem aminoxylation/O-N bond heterolysis reactions. Org. Lett..

[71] Joseph J., Ramachary D.B., Jemmis E.D. (2006). Electrostatic repulsion as an additional selectivity factor in asymmetric proline catalysis. Org. Biomol. Chem..

[72] Font D., Bastero A., Sayalero S., Jimeno C., Pericàs M.A. (2007). Highly enantioselective α-aminoxylation of aldehydes and ketones with a polymer-supported organocatalyst. Org. Lett..

[73] Huang K., Huang Z.-Z., Li X.-L. (2006). Highly enantioselective α-aminoxylation of aldehydes and ketones in ionic liquids. J. Org. Chem..

[74] Guo H.-M., Niu H.-Y., Xue M.-X., Guo Q.-X., Cun L.-F., Mi A.-Q., Jiang Y.-Z., Wang J.-J. (2006). L-Proline in an ionic liquid as an efficient and reusable catalyst for direct asymmetric α-aminoxylation of aldehydes and ketones. Green Chem..

[75] Cheong P. H.-Y., Houk K.N. (2004). Origins of selectivities in proline-catalyzed α-aminoxylations. J. Am. Chem. Soc..

[76] Mathew S.P., Iwamura H., Blackmond D.G. (2004). Amplification of enantiomeric excess in a proline-mediated reaction. Angew. Chem. Int. Ed..

[77] Poe S.L., Bogdan A.R., Mason B.P., Steinbacher J.L., Opalka S.M., McQuade D.T. (2009). Use of bifunctional ureas to increase the rate of proline-catalyzed α-aminoxylations. J. Org. Chem..

[78] Zotova N., Moran A., Armstrong A., Blackmond D.G. (2009). A coherent mechanistic rationale for additive effects and autoinductive behaviour in proline-mediated reactions. Adv. Synth. Catal..

[79] Hayashi Y., Yamaguchi J., Hibino K., Sumiya T., Urushima T., Shoji M., Hashizume D., Koshino H. (2004). A highly active 4-siloxyproline catalyst for asymmetric synthesis. Adv. Synth. Catal..

[80] Wang W., Wang J., Li H., Liao L. (2004). An amine sulfonamide organocatalyst for promoting direct, highly enantioselective α-aminoxylation reactions of aldehydes and ketones. Tetrahedron Lett..

[81] Chua P.J., Tan B., Zhong G. (2009). Highly enantioselective L-thiaproline catalyzed α-aminoxylation of aldehydes in aqueous media. Green Chem..

[82] Sundén H., Dahlin N., Ibrahem I., Adolfsson H., Córdova A. (2005). Novel organic catalysts for the direct enantioselective α-oxidation of carbonyl compounds. Tetrahedron Lett..

[83] Kano T., Yamamoto A., Mii H., Takai J., Shirakawa S., Maruoka K. (2008). Direct asymmetric aminoxylation reaction catalyzed by axially chiral amino acids. Chem. Lett..

[84] Kano T., Yamamoto A., Maruoka K. (2008). Direct asymmetric aminoxylation reaction catalyzed by a binaphthyl-based chiral amino sulfonamide with high catalytic performance. Tetrahedron Lett..

[85] Kano T., Yamamoto A., Shirozu F., Maruoka K. (2009). Enantioselectivity switch in direct asymmetric aminoxylation catalyzed by binaphthyl-based chiral secondary amines. Synthesis.

[86] Lu M., Lu Y., Zeng X., Tan B., Xu Z., Zhong G. (2009). Chiral Brønsted acid-catalyzed enantioselective α-hydroxylation of β-dicarbonyl compounds. J. Am. Chem. Soc..

[87] Zhong G. (2004). Tandem aminoxylation-allylation reactions: a rapid, asymmetric conversion of aldehydes to mono-substituted 1,2-diols. Chem. Commun..

[88] Zhong G., Yu Y. (2004). Enantioselective synthesis of allylic alcohols by the sequential aminoxylation-olefination reactions of aldehydes under ambient conditions. Org. Lett..

[89] Yang L., Liu R.-H., Wang B., Weng L.-L., Zheng H. (2009). Asymmetric synthesis of 3-hydroxyl-2-alkanones *via* tandem organocatalytic aminoxylation of aldehydes and chemoselective diazomethane homologation. Tetrahedron Lett..

[90] Kumarn S., Shaw D.M., Longbottom D.A., Ley S.V. (2005). A highly selective, organocatalytic route to chiral dihydro-1,2-oxazines. Org. Lett..

[91] Kumarn S., Shaw D.M., Ley S.V. (2006). A highly selective, organocatalytic route to chiral 1,2-oxazines from ketones. Chem. Comm..

[92] Kumarn S., Oelke A.J., Shaw D.M., Longbottom D.A., Ley S.V. (2007). A sequential enantioselective, organocatalytic route to chiral 1,2-oxazines and chiral pyridazines. Org. Biomol. Chem..

[93] Yamamoto Y., Momiyama N., Yamamoto H. (2004). Enantioselective tandem *O*-nitroso aldol/Michael reaction. J. Am. Chem. Soc..

[94] Momiyama N., Yamamoto Y., Yamamoto H. (2007). Diastereo- and enantioselective synthesis of nitroso Diels-Alder-type bicycloketones using dienamine: Mechanistic insight into sequential nitroso aldol/Michael reaction and application for optically pure 1-amino-3,4-diol synthesis. J. Am. Chem. Soc..

[95] Zhu D., Lu M., Chua P.J., Tan B., Wang F., Yang X., Zhong G. (2008). A highly stereoselective organocatalytic tandem aminoxylation/aza-Michael reaction for the synthesis of tetrahydro-1,2-oxazines. Org. Lett..

[96] Lu M., Zhu D., Lu Y., Hou Y., Tan B., Zhong G. (2008). Organocatalytic asymmetric α-aminoxylation/aza-Michael reactions for the synthesis of functionalized tetrahydro-1,2-oxazines. Angew. Chem. Int. Ed..

[97] Zhu D., Lu M., Dai L., Zhong G. (2009). Highly stereoselective one-pot synthesis of bicyclic isoxazolidines with five stereogenic centers by an organocatalytic process. Angew. Chem. Int. Ed..

[98] Kondekar N.B., Kumar P. (2009). Iterative approach to enantiopure *syn/anti*-1,3-polyols *via* proline-catalyzed sequential α-aminoxylation and Horner-Wadsworth-Emmons olefination of aldehydes. Org. Lett..

[99] Enders D., Müller M. (2004). Efficient asymmetric syntheses of (+)-strictifolione. Synthesis.

[100] George S., Sudalai A. (2007). Enantioselective synthesis of tarchonanthuslactone using proline-catalyzed asymmetric α-aminooxylation. Tetrahedron Asymmetry.

[101] Mangion I.K., MacMillan D.W.C. (2005). Total synthesis of brasoside and littoralisone. J. Am. Chem. Soc..

[102] Yamaguchi J., Toyoshima M., Shoji M., Kakeya H., Osada H., Hayashi Y. (2006). Concise enantio- and diastereoselective total syntheses of fumagillol, RK-805, FR65814, ovalicin, and 5-demethylovalicin. Angew. Chem. Int. Ed..

[103] Varseev G.N., Maier M.E. (2007). Enantioselective total synthesis of (+)-neosymbioimine. Org. Lett..

[104] Hara S., Makino K., Hamada Y. (2006). Total synthesis of halipeptin A, a potent anti-inflammatory cyclodepsipeptide from a marine sponge. Tetrahedron Lett..

[105] Kim S.-G., Park T.-H., Kim B. J. (2006). Efficient total synthesis of (+)-exo-, (-)-endo-brevicomin and their derivatives *via* asymmetric organocatalysis and olefin cross-metathesis. Tetrahedron Lett..

[106] Narina S.V., Sudalai A. (2006). Short and practical enantioselective synthesis of linezolid and eperezolid *via* proline-catalyzed asymmetric α-aminooxylation. Tetrahedron Lett..

[107] Kotkar S.P., Sudalai A. (2006). A short enantioselective synthesis of the antiepileptic agent, levetiracetam based on proline-catalyzed asymmetric α-aminooxylation. Tetrahedron Lett..

[108] Kotkar S.P., Sudalai A. (2006). Enantioselective synthesis of (*S*,*S*)-ethambutol using proline-catalyzed asymmetric α-aminooxylation and α-amination. Tetrahedron Asymmetry.

[109] Kotkar S.P., Suryavanshi G.S., Sudalai A. (2007). A short synthesis of (+)-harzialactone A and (*R*)-(+)-4-hexanolide *via* proline-catalyzed sequential α-aminooxylation and Horner-Wadsworth-Emmons olefination of aldehydes. Tetrahedron-Asymmetr.

[110] Talluri S.K., Sudalai A. (2007). An organo-catalytic approach to the enantioselective synthesis of (*R*)-selegiline. Tetrahedron.

[111] Panchgalle S.P., Gore R.G., Chavan S.P., Kalkote U.R. (2009). Organocatalytic enantioselective synthesis of β-blockers: (*S*)-propranolol and (*S*)-naftopidil. Tetrahedron-Asymmetr..

[112] Kim S.-G. (2009). Concise total synthesis of (+)-disparlure and its *trans*-isomer using asymmetric organocatalysis. Synthesis.

[113] Sawant R.T., Waghmode S.B. (2009). Organocatalytic enantioselective formal synthesis of HRV 3C-protease inhibitor (1*R*,3*S*)-thysanone. Tetrahedron.

[114] Kim S.-G., Park T.-H. (2006). Organocatalyzed asymmetric α-hydroxyamination of α-branched aldehydes: Asymmetric synthesis of optically active *N*-protected α,α-disubstituted amino aldehydes and amino alcohols. Tetrahedron Lett..

[115] Guo H.-M., Cheng L., Cun L.-F., Gong L.-Z., Mi A.-Q., Jiang Y.-Z. (2006). L-Prolinamide-catalyzed direct nitroso aldol reactions of α-branched aldehydes: A distinct regioselectivity from that with L-proline. Chem. Commun..

[116] Palomo C., Vera S., Velilla I., Mielgo A., Gómez-Bengoa E. (2007). Regio- and enantioselective direct oxyamination reaction of aldehydes catalyzed by α,α-diphenylprolinol trimethylsilyl ether. Angew. Chem. Int. Ed..

[117] Kano T., Ueda M., Takai J., Maruoka K. (2006). Direct asymmetric hydroxyamination reaction catalyzed by an axially chiral secondary amine catalyst. J. Am. Chem. Soc..

[118] Momiyama N., Yamamoto H. (2005). Brønsted acid catalysis of achiral enamine for regio- and enantioselective nitroso aldol synthesis. J. Am. Chem. Soc..

[119] López-Cantarero J., Cid M.B., Poulsen T.B., Bella M., Ruano J.L.G., Jørgensen K.A. (2007). Intriguing behavior of cinchona alkaloids in the enantioselective organocatalytic hydroxyamination of α-substituted-α-cyanoacetates. J. Org. Chem..

[120] Sibi M.P., Hasegawa M. (2007). Organocatalysis in radical chemistry. Enantioselective α-oxyamination of aldehydes. J. Am. Chem. Soc..

[121] Bui N.-N., Ho X.-H., Mho S.I., Jang H.-Y. (2009). Organocatalyzed α-oxyamination of aldehydes using anodic oxidation. Eur. J. Org. Chem..

[122] Vaismaa M.J.P., Yau S.C.Y., Tomkinson N.C.O. (2009). Organocatalytic α-oxybenzoylation of aldehydes. Tetrahedron Lett..

[123] Gotoh H., Hayashi Y. (2009). Diphenylprolinol silyl ether as a catalyst in an asymmetric, catalytic and direct α-benzoyloxylation of aldehydes. Chem. Commun..

[124] Kano T., Mii H., Maruoka K. (2009). Direct asymmetric benzoyloxylation of aldehyde catalyzed by 2-tritylpyrrolidine. J. Am. Chem. Soc..

[125] Ibrahem I., Zhao G.-L., Sundén H., Córdova A. (2006). A route to 1,2-diols by enantioselective organocatalytic α-oxidation with molecular oxygen. Tetrahedron Lett..

[126] Córdova A., Sundén H., Engqvist M., Ibrahem I., Casas J. (2004). The direct amino acid-catalyzed asymmetric incorporation of molecular oxygen to organic compounds. J. Am. Chem. Soc..

[127] Acocella M.R., Mancheño O.G., Bella M., Jørgensen K.A. (2004). Organocatalytic asymmetric hydroxylation of β-keto esters: Metal-free synthesis of optically active anti-diols. J. Org. Chem..

[128] Bekele T., Shah M.H., Wolfer J., Abraham C.J., Weatherwax A., Lectka T. (2006). Catalytic, enantioselective [4+2]-cycloadditions of ketene enolates and *o*-quinones: efficient entry to chiral, α-oxygenated carboxylic acid derivatives. J. Am. Chem. Soc..

[129] Hernandez-Juan F.A., Cockfield D.M., Dixon D.J. (2007). Enantioselective organocatalytic aryloxylation of aldehydes with *o*-quinones. Tetrahedron Lett..

[130] Engqvist M., Casas J., Sundén H., Ibrahem I., Córdova A. (2005). Direct organocatalytic asymmetric α-oxidation of ketones with iodosobenzene and *N*-sulfonyloxaziridines. Tetrahedron Lett..

[131] Tong A.-T., Brimble M.A., Barker D. (2009). Influence of α-methyl substitution of proline-based organocatalysts on the asymmetric α-oxidation of aldehydes. Tetrahedron.

[132] Richardson R.D., Page T.K., Altermann S., Paradine S.M., French A.N., Wirth T. (2007). Enantioselective α-oxytosylation of ketones catalysed by iodoarenes. Synlett.

[133] Altermann S.M., Richardson R.D., Page T.K., Schmidt R.K., Holland E., Mohammed U., Paradine S.M., French A.N., Richter C., Bahar A.M., Witulski B., Wirth T. (2008). Catalytic enantioselective α-oxysulfonation of ketones mediated by iodoarenes. Eur. J. Org. Chem..

